# Implementation of Matrix-Matched Semiquantification of PFAS in AFFF-Contaminated Soil

**DOI:** 10.1021/acs.est.4c14255

**Published:** 2025-04-03

**Authors:** Catharina Capitain, Melanie Schüßler, Boris Bugsel, Jonathan Zweigle, Christian Vogel, Peter Leube, Christian Zwiener

**Affiliations:** † Environmental Analytical Chemistry, Department of Geosciences, University of Tübingen, Schnarrenbergstraße 94-96, 72076 Tübingen, Germany; ‡ Department of Plant and Environmental Sciences, University of Copenhagen, Thorvaldsensvej 40, Frederiksberg, Kobenhavn 1871, Denmark; § Federal Institute for Materials Research and Testing, Division 4.4 − Thermochemical Residues Treatment and Resource Recovery, Unter den Eichen 87, 12205 Berlin, Germany

**Keywords:** PFAS, AFFF, soil, HRMS, semiquantification

## Abstract

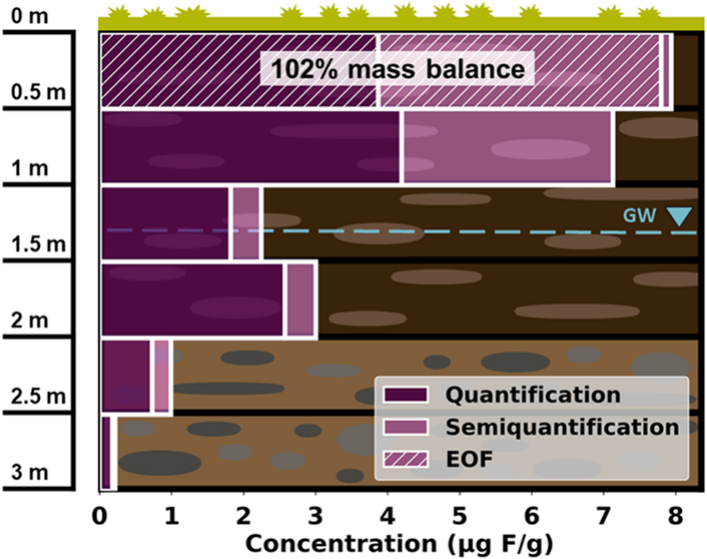

This study presents a novel semiquantification approach for nontarget screening (NTS), combining matrix-matched calibration and ionization class-specific average calibration curves (ACCs) to address the lack of analytical reference standards for most per- and polyfluoroalkyl substances (PFAS). Ionization class-specific ACCs for carboxylic and sulfonic acids, sulfonamides, and cationic PFAS result in high accuracy, with median absolute accuracy quotients below 2.27×. The approach was applied to soil impacted by aqueous film-forming foam (AFFF) contamination. A total of 96 tentatively identified PFAS were semiquantified in addition to 28 quantified compounds based on available standards. Semiquantified concentrations exceeded those of target analytes, demonstrating the critical role of this method in capturing broader PFAS contamination. In this case, validation against extractable organofluorine (EOF) showed a 102% closed mass balance. The innovative approach not only enables comprehensive PFAS contamination assessment in complex matrices but also expands the scope of the NTS for environmental monitoring, remediation, and risk assessment of AFFF-contaminated sites.

## Abstract 2

Improved semiquantification of AFFF PFAS in soil reveals high concentrations of tentatively identified PFAS, highlighting the importance of a robust semiquantification method.

## Introduction

Per- and polyfluoroalkyl substances (PFAS) have been increasingly detected in soil,^1−5^ groundwater,^5−8^ drinking water,^9−11^ wildlife,^12−14^ and human blood,^10,15−17^ even in remote regions such as the arctic^18−21^ in the past decade. The ubiquitous distribution of PFAS is cause for concern due to their extreme stability in environmental compartments^22−24^ and adverse health effects such as liver damage, kidney and testicular cancer, lower birthweight, and thyroid disease.^25^

More than 200 potential applications in industry and consumer products have been identified for PFAS, including as active ingredients in aqueous film-forming foams (AFFF).^26^ PFAS employed in AFFF can be of anionic, cationic, or zwitterionic nature and vary in structure and functional groups.^6,8,27−29^ Due to the versatile structures of AFFF PFAS, various names for the same substance exist in the literature. In this study, we applied the novel terminology system introduced by Schüßler et al.^30^

AFFF formulations contain about 2–15% of fluorosurfactants.^31^ Therefore, during firefighting activities employing AFFF, large amounts of PFAS can enter the environment and, ultimately, groundwater. In 2008, an agricultural area in Reilingen, Germany, was highly contaminated with AFFF PFAS during firefighting operations. An extended characterization of the PFAS contamination pattern in Reilingen by liquid chromatography–high-resolution mass spectrometry (LC-HRMS) was done by Schüßler et al.^30^ 124 PFAS from 42 PFAS subclasses could be identified in soil from the Reilingen site after successful data prioritization by the novel MD/C-m/C approach^32^ (mass defect per carbon number (MD/C) plotted against mass per carbon number (m/C)) and the recently developed software tool PFΔ*Screen*.^33^ Since only 28 of the 124 identified PFAS could be quantified based on analytical standards, semiquantification approaches have to be applied to assess the extent of contamination and to propose suitable remediation measures.

Typically, for semiquantification, a structurally similar substance is selected as a surrogate for the target PFAS for a 1:1 matching procedure. Thereby, the molar response factor (RF) from the surrogate, which is the quotient of a measured chemical signal (e.g., the integrated peak area) and a known concentration, is applied to the suspect PFAS. However, this method requires suitable standards, is time-consuming, and often lacks accuracy, especially when the substances are structurally diverse as in the case of AFFF contamination.

To meet these challenges, Cao et al.^34^ developed average calibration curves (ACCs) for cationic and anionic PFAS, respectively. These calibration curves were fitted with log–log and weighted linear regression models and used to semiquantify the suspect PFAS concentrations. In another promising approach, Pu et al.^35^ used bootstrap-sampled calibration values from chemical surrogates for semiquantification, demonstrating that the selection of ‘expert-selected’ surrogates improved predictive accuracy compared to the use of all available surrogates. Another approach is to predict ionization efficiencies in electrospray ionization using a set of molecular predictors and machine learning for semiquantification.^36^ An interlaboratory comparison of five quantification approaches for a broad range of analyte structures and properties revealed the best performance for an ionization efficiency prediction approach with a mean prediction error of 15×.^37^

In quantification and semiquantification, it is also important to account for matrix effects (MEs) which is typically done very accurately with the corresponding isotope-labeled standards. However, labeled PFAS standard availability is very limited to a few anionic PFAS. No cationic or zwitterionic labeled PFAS standards were available at the time of this study. Alternatively, the standard addition method can be applied which involves several steps of analyte spikes to an unknown sample and very accurately accounts for ME from the sample matrix during quantification.^38^

Traditional targeted analytical methods focus on a limited set of known PFAS, potentially overlooking numerous unidentified or emerging variants present in the environment. To address this limitation, integrating semiquantitative nontarget screening (NTS) techniques is essential. These approaches enable the detection and estimation of concentrations for a broader spectrum of PFAS, providing a more comprehensive assessment of environmental contamination.^2,39^ The objective of this study was therefore to implement a matrix-matched semiquantification approach for AFFF PFAS and their transformation products (TPs), to quantitatively assess the AFFF contamination, and to include a fluorine mass balance based on extractable organofluorine (EOF) measurements. 39 PFAS standards have been subdivided into four subclasses, carboxylic and sulfonic acids, sulfonamides, and cationic/zwitterionic PFAS, and used for matrix-matched class-specific ACCs. Semiquantification was then performed for 96 tentatively identified PFAS in soil samples from the contaminated site. The fluorine mass balance was finally compared to the EOF measurements.

## Materials and Methods

### Chemicals and Reagents

All chemicals were of LC-MS grade. Ammonium acetate (NH_4_Ac), methanol (MeOH), and water were purchased from Thermo Fisher Scientific. For detailed information on the 51 authentic PFAS reference standards and the mass-labeled internal standards (IS) that were used for quantification, refer to the Supporting Information (SI, Tables S1 and S2).

### Sampling and Processing of Soil Samples

Soil samples from the *Nachtwaidgraben* field site in Reilingen were taken in May 2023, as previously reported.^30^ Shortly, at location *S1* four drill cores on a 1 × 1 m^2^ (*S1–1, S1–2, S1–3, S1–4*), each reaching up to a depth of 3 m, were acquired (Figure S1). The drill cores were vertically divided into six sections, each measuring 0.5 m. Six composite depth section samples from the four drill cores were then prepared (*S1L1*: 0–0.5, *S1L2*: 0.5–1, *S1L3*: 1–1.5, *S1L4*: 1.5–2, *S1L5*: 2–2.5, *S1L6*: 2.5–3 m). Composite depth section samples were dried (40 °C), ground, and sieved (≤1.6 mm). 5 g soil per composite depth section sample was sequentially extracted three times with MeOH + 0.4 M NH_4_Ac as reported elsewhere.^30^ The final combined extracts were then used for all further analyses, including EOF measurements. To assess the recovery, the extraction method was tested on standard soil (LUFA SP6S, ∼1.55% organic carbon) spiked with the PFAS standard mixture. MEs were determined using IS spike, and extraction recoveries of compounds with available IS were corrected accordingly. Extraction efficiencies were further compared to sequential extraction with pure MeOH. Extraction blanks consisting of pure extraction solvent were processed the same way as the soil samples to check for background contamination during the extraction process.

### Soil Characteristics

Total organic carbon (TOC) and cation exchange capacity (CEC) were determined for all depth sections. For TOC measurements, samples were additionally ground in an oscillating mill (MM400, Retsch, Germany) for 60 s at 20 Hz and analyzed for TOC with a SoliTOC Cube (Elementar, Germany), following DIN 19539.

CEC was determined with a modified NH_4_Ac method at pH 8.5 as 5 of 12 soil samples had a pH > 7.4, indicating the presence of calcareous minerals.^40,41^ 1.2 g of soil was weighed in a polypropylene (PP) tube, and 20 mL of 1 M NH_4_Ac (pH 8.5) extraction solution was added. Samples were then shaken for 1 h, centrifuged for 10 min at 7830 relative centrifugal force (rcf), and the supernatant was filtered (0.2 μm, PES Agilent Captiva syringe filters). Extracts were stored at 4 °C, diluted (1:2, 1:10, and 1:100), and acidified (2% HNO_3_) prior to analysis. The extracts were analyzed for Ca^2+^, Mg^2+^, Na^+^, and K^+^ at 393.366, 518.360, 588.995, and 766.491 nm, respectively, by microwave plasma–atomic emission spectroscopy (MP-AES, 4200 MPAES, Agilent Technologies, Australia). CEC was determined according to eq 1, where *n* is the amount of the respective cation in moles and *m* is the mass of soil in grams
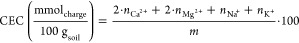
1

### Instrumental Analysis and Data Evaluation

Analysis of soil extracts, extraction blank, MeOH blank, and quality controls (QCs) was performed using a high-performance liquid chromatography (1290 HPLC from Agilent Technologies, Waldbronn, Germany) coupled to a quadrupole time-of-flight mass spectrometer with an electrospray ionization source (6550 QTOF, Agilent Technologies, Santa Clara, HPLC-ESI-QTOF-MS). An Agilent C_18_ column (Poroshell 120 EC-C_18_, 2.1 × 100 mm, particle size 2.7 μm) was used for separation. Gradient elution with a total runtime of 22 min (chromatographic details in Table S3) with eluent A (95/5 H_2_O/MeOH + 2 mM NH_4_Ac) and eluent B (95/5 MeOH/H_2_O + 2 mM NH_4_Ac) was employed.

Both ionization modes (ESI*–*/ESI+) were applied separately. The QTOF was operated in data-dependent MS^2^ mode (ddMS^2^) with an acquisition rate of 3 spectra/s (MS^1^ and MS^2^). A precursor was selected for MS^2^ if a threshold of 1000 counts was exceeded and was excluded for the next 0.5 min after recording 3 MS^2^ spectra. The applied collision energy (CE) was determined according to eq 2, where *m*/*z* is the mass to charge ratio. This equation was optimized for PFAS standards and demonstrated optimal collision behavior for these compounds
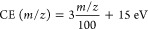
2The 124 identified confidence level 1–3 substances from Schüßler et al.^30^ were quantified (confidence level 1) or semiquantified (confidence level 2–3). A more detailed description of the identification of these PFAS and the assignment of the confidence levels can be found elsewhere.^30^ The relative standard deviation (RSD) of replicate measurements was better than 5% (*n* = 5). Samples for quality control (QCs) were analyzed after every eighth sample and confirmed acceptable results with negligible instrument drift (±10%). No contaminants were detected in the extraction or measurement blanks.

### Quantification and Semiquantification of PFAS in Topsoil (*S1L1*)

The uppermost soil extract of *S1L1* (topsoil) was chosen to set up the quantification and semiquantification model due to the highest peak intensities in this sample. Due to the lack of commercially available mass-labeled internal standards, the basis for both models (quantification and semiquantification) is a standard addition approach in order to account for ME. For quantification (target PFAS, Table S1), standard addition was performed, using seven calibration points in the range of 0.1–10 μg/L in positive and negative ionization mode, respectively. Spiking of the respective volumes of PFAS standard mix into the sample was performed by the autosampler, mixing the respective proportions of PFAS standard mix, dilution solution (MeOH), and soil extract in-needle (total injection volume 10 μL). Diluted samples (1:20, 1:100, 1:200) were also spiked using the same procedure as the undiluted extracts, resulting in up to four calibration curves for each compound.

The limit of quantification (LOQ) was defined as the lowest measured standard for which an S/N ratio >10 was found. The MassHunter Qualitative Analysis 10.0 software was used to detect and integrate suspect peaks with the FindbyFormula algorithm (accuracy of ±10 ppm). The response (area) was calculated from the following ion species: [M-H]^−^ for anionic PFAS and [M + H]^+^, [M + Na]^+^, [M + K]^+^, [M + NH_4_]^+^, and [M + C_2_H_7_N_2_]^+^ (acetonitrile + NH_4_) for cationic PFAS with neutral mass [M], as well as all detected isotopes. Neutral losses were not included due to false assignments by the software and to ensure a consistent approach. Consequently, even well-known neutral losses, such as [M-CO_2_–H]^−^ for perfluoroalkyl carboxylic acids (PFCAs), were not considered to avoid systematically underestimating tentatively identified PFAS. Linear and branched isomers were integrated and (semi)quantified together. Responses were exported and processed using an in-house developed Python code.

For the semiquantification model of suspect PFAS (Table S4), standard addition was applied as described before, in order to account for ME. Average calibration curves (ACC) were created via log–log transformation of the molar concentrations, and the responses of analytical reference standards were similar to those described by Cao et al.^34^ In detail, the intercepts of the calibration curves of each standard (responses from soil sample) were subtracted, and the molar concentration and the responses were logarithmized (log–log transformation). Then, a linear regression of all data points was conducted (calibration curves of perfluorooctanesulfonic acid (PFOS), 6:2 fluorotelomer sulfonamide propyl betaine (FTSAm-Pr-B), and 5:1:2 fluorotelomer betaine (FTB) in the undiluted or less diluted extract matrices were omitted due to a too high concentration of the analyte in the sample itself, which distorts the calibration curves). To improve the accuracy of semiquantification, for each class of functional groups (carboxylic acids, sulfonic acids, sulfonamides, and cationic plus zwitterionic PFAS), a separate ACC was constructed. The four ACCs represent all available standards (Table S5). The ACCs were used to reestimate the concentration levels of the 39 PFAS standards. Absolute accuracy quotients (AAQs) were determined according to eq 3, where *C*_semiquant_ is derived from ACCs and *C*_quant_ is derived by calibration with standard additions of the respective analytical reference standards. Data points with high AAQs (>20) were manually reviewed and considered as outliers since they resulted from systematic errors (19 out of 852 data points).
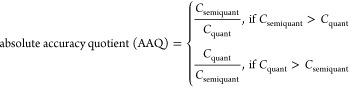
3The linear regressions were repeated without outliers to obtain the final four ACCs for the four classes of functional groups. These ACCs were used to perform semiquantification of 96 tentatively identified PFAS (levels 2 and 3 PFAS) which were subdivided also into the 4 ionization classes (Figure S2). If a PFAS contained several functional ionization moieties, it was semiquantified with the ACC with the lowest median AAQ, assuming the lowest error. If necessary, compounds were semiquantified at a 1:200 dilution to ensure that the response was within the calibration range of the applied ACC.

### Semiquantification of Other Soil Depths

The matrix-matched ACCs specifically considered the ME of the topsoil extracts. To assess the applicability of these ACCs to soil samples from different depths (*S1L2–S1L6*), MEs at different depths were determined using an IS spike (4 μg/L, respectively, Table S2) and calculated according to eq 4, where area IS_extract_ is the peak area of the IS spiked to the soil extract and area IS_extract topsoil_ is the peak area of the IS spiked to the topsoil extract
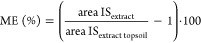
4Due to instrumental drift of the QTOF between different batches, the peak area was corrected using a factor based on peak area of topsoil from both measurements for each compound. The correction factors were between 0.5 and 5.1, but only 6 out of 124 substances had a factor exceeding 2 (mean: 1.1). The calibration curves of individual PFAS standards for quantification, as well as the constructed ACCs for semiquantification, were applied to the different depths, as described above. Concentrations of single PFAS had to be corrected due to peak saturation or concentrations outside the calibration range based on the deviation of the peak area of the undiluted sample compared to that of the diluted sample.

### EOF

Extractable organofluorine (EOF) was determined for the top layer of soil (S1L1) by combustion ion chromatography (CIC). 250 μL of extract were injected and combusted in the induction furnace (AQF-2100H, GA-210, Mitsubishi Chemical Analytech, Tokyo, Japan) at 1050 °C under a flow of O_2_ (300 mL/min) and Ar (150 mL/min). Combustion gases were absorbed in a freshly prepared 150 μmol/L NH_3_ absorption solution with methyl sulfonate (∼2 mg/L) as an internal standard in the absorption unit. 100 μL of the absorption solution was then analyzed by ion chromatography (IC; ICS Integrion, Thermo Fisher Scientific, Dreieich, Germany) on a Dionex IonPac AS20 (2 × 250 mm^2^) separation column with a KOH gradient program. Fluoride ions were detected by suppressed conductivity using a dynamically regenerated suppressor (Dionex ADRS 600). A nine-point calibration with NH_4_F at levels between 44 and 5000 μg F/L (*R*^2^ = 0.9994) was used for quantification. All EOF samples were measured in triplicates. More details are given in the SI (Tables S6–S9).

## Results and Discussion

### Method Validation

#### Topsoil

**Figure 1:**
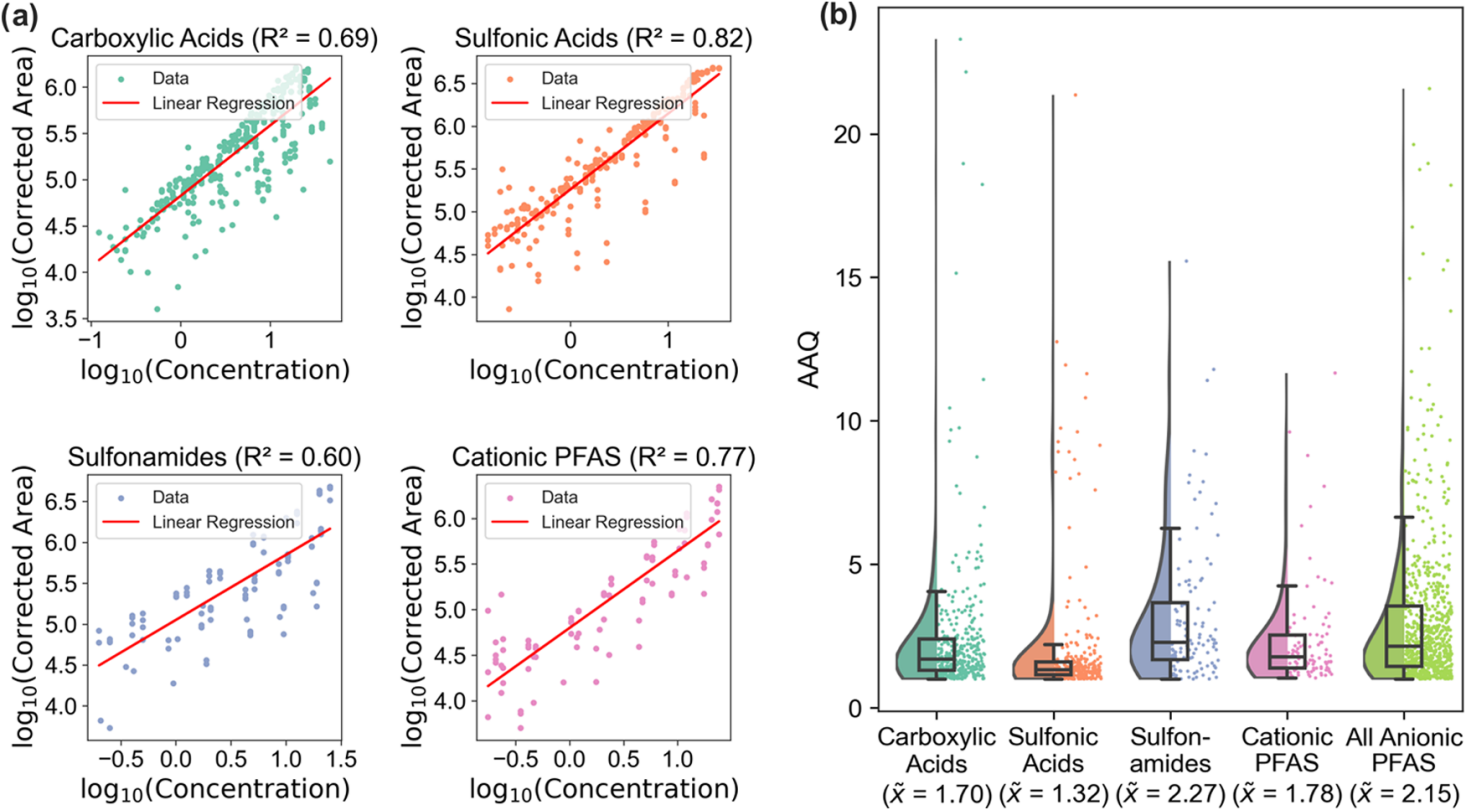
(a) Matrix-matched average calibration curves (ACCs) for four ionization classes based on 39 authentic standards at 7 concentration levels and 4 matrix dilutions (dots represent measured data, lines represent linear regression). (b) Absolute accuracy quotients (AAQ) of the semiquantification of standards for the four ionization classes and all anionic PFAS (box plots with median, 25 and 75 percentile and violin plots).

Semiquantification was performed using an ACC approach coupled with the standard addition method to consider ME and therefore improve the quantification accuracy. Distinct ACCs for four ionization classes of PFAS were constructed: cationic and zwitterionic compounds, carboxylic acids, sulfonic acids, and sulfonamides with 5, 21, 12, and 6 analytical reference standards, respectively (Figure 1a and Table S5). The four ACCs were used to reestimate the concentrations of 39 PFAS standards for 4 matrix dilutions at 7 concentration levels, which resulted in 28 data points for each PFAS standard. The AAQ was calculated using the quantified and the semiquantified concentration for each data point, yielding median values for cationic and zwitterionic PFAS, carboxylic acids, sulfonic acids, and sulfonamides of 1.78× (90th percentile = 4.3, *n* = 5), 1.70× (90th percentile = 4.0, *n* = 21), 1.32× (90th percentile = 2.5, *n* = 12), and 2.27× (90th percentile = 6.8, *n* = 6), respectively (Figure 1b). If all available anionic PFAS standards were used together for one ACC (*n* = 37), the median AAQ would be 2.15× (90th percentile = 5.7) and the variability would be greater compared to the separate ACCs for the different ionization classes (Figures 1b and S3). This clearly demonstrates that concentrations can be semiquantified more accurately, taking the different ionization classes into consideration. The median AAQ and the standard deviation are higher for sulfonamides, which is a result of the large structural variety of only 6 sulfonamide standards and hence a large range of RFs. Compared to the literature, the AAQs determined in this work are rather low (Table S10), proving the approach of matrix-matched ACCs for specific ionization classes of PFAS viable.^34−37^

#### All Soil Depths

To assess whether the matrix-matched ACCs for the topsoil are also applicable to other soil depths, the ME of different depths relative to the topsoil were evaluated with IS spikes. In general, most of the mass-labeled IS showed signal enhancement in the lower soil compared to the topsoil, while for short-chain PFCAs (perfluoropentanoic acid (PFPeA), perfluorohexanoic acid (PFHxA)), the signal was suppressed. The MEs of all isotopically labeled PFCAs and perfluoroalkyl sulfonic acids (PFSAs) ranged from −40 to 35% (Figure S4), with the range for individual substances varying between 15 and 40%, except for perfluorononanoic acid (PFNA) and PFOS. The reason for these two compounds deviating significantly in the topsoil is due to signal suppression in the upper soil layers, as PFOS is present in very high concentrations in these layers and coelutes with PFNA, but also to signal enhancement in deeper soil layers. As mentioned before, substances with excessively high concentrations in the sample, such as PFOS, were therefore excluded from the ACCs. Since all other compounds exhibited MEs in an acceptable range for semiquantification and the fact that ACCs are also based on diluted samples with even lower MEs, the ACCs can be confidently applied to the other depth sections without any adaptation. Minor inaccuracies (<40%) had to be accepted, which, however, fall within the typical error range of semiquantification (Table S10).

### PFAS Concentrations in Topsoil

**Figure 2:**
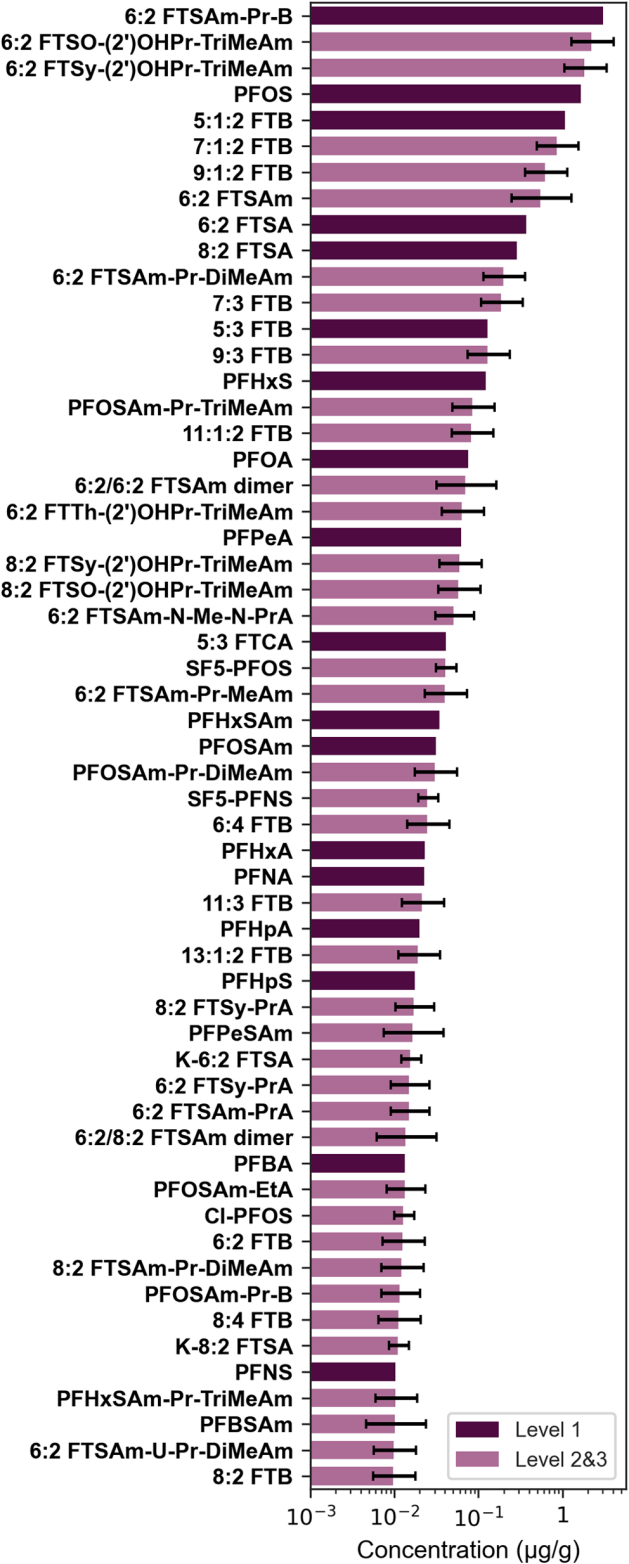
Concentrations of quantified (dark purple) and semiquantified (light purple) PFAS in the extract of topsoil on site *S1L1* in μg/g displayed with a logarithmic scale. Only compounds with concentrations ≥10^–2^ μg/g are shown (all compounds in Figure S5). Error bars for semiquantified PFAS represent the median AAQ of the corresponding ionization class (compound names and structures in Table S4 and concentrations in Table S11).

PFAS concentrations were finally semiquantified for the topsoil (topsoil, *S1L1*) with matrix-matched ACCs of the four ionization classes for 96 tentatively identified AFFF PFAS resulting in a sum concentration of 7.86 μg/g (Figures 2, S5, and Table S11). Based on the ACC for cationic PFAS, 36 substances were semiquantified with a sum concentration of 6.83 μg/g. The most abundant cationic AFFFs are 6:2 fluorotelomer sulfoxide propanol trimethylamine (FTSO-(2′)OHPr-TriMeAm, 2.29 μg/g), 6:2 fluorotelomer sulfone propanol trimethylamine (FTSy-(2′)OHPr-TriMeAm, 1.88 μg/g), 7:1:2 FTB (0.88 μg/g), and 9:1:2 FTB (0.64 μg/g). With the sulfonamide-based ACC 6:2 fluorotelomer sulfonamide (FTSAm, 0.57 μg/g) and 9 further AFFF PFAS at a sum concentration of 0.69 μg/g were determined. In the case of anionic PFAS 8 carboxylic acids (sum of 0.14 μg/g) and 42 sulfonic acids (sum of 0.21 μg/g) were semiquantified. The results reveal the dominant abundance of cationic PFAS with 87% semiquantified in the topsoil (Table S11).

Based on matrix-matched standard calibration with authentic standards, 28 PFAS were quantified in the topsoil (Figures 2, S5, and Table S11). The highest concentrations were observed for the AFFF constituents PFOS (1.70 μg/g), 6:2 FTSAm-Pr-B (3.17 μg/g), and 5:1:2 FTB (1.11 μg/g), and the sum concentration is 7.33 μg/g. Also, cationic PFAS dominated with approximately 60%.

This pattern, characterized by many precursors and a high proportion of cationic PFAS, is typical of AFFF-contaminated sites. However, this contaminated soil exhibits a particularly diverse composition of PFAS, as different fire stations were involved, and thus foams from various manufacturers were applied.^5,30,42^

The total sum concentration of quantified and semiquantified PFAS is 15.19 μg/g or on a fluorine basis, 7.96 μg F/g. Comparison to the measured EOF of 7.8 μg F/g reveals a completely closed fluorine mass balance for the topsoil, which is a somehow surprising result considering the limited accuracy of the semiquantified PFAS. Nevertheless, the results show the importance of nontarget screening and semiquantification for site characterization.

Similar high concentrations have been found at other AFFF-contaminated sites, such as in the study by Bigler et al.,^43^ which reported concentrations of 31 μg/g, Nickerson et al.,^5^ who found up to 3.81 μg/g, at different firefighter training areas, or others.^44^

**Figure 3:**
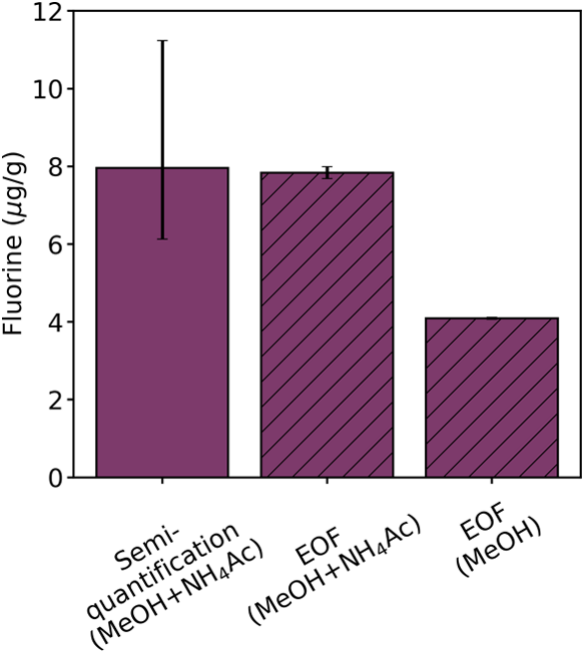
Equivalents of fluorine found by the semiquantification approach (extraction with MeOH + NH_4_AC) and by EOF measurements after extractions with MeOH + NH_4_Ac and with pure MeOH (error bars represent the median AAQs of the corresponding ionization classes for the semiquantification and the standard deviations for EOF measurements).

Since a dominant fraction of the PFAS contamination in the topsoil of 74% was due to cationic PFAS, the importance of addition of ammonia to the extraction solvent was of particular interest. For that, EOF in extracts from extraction with pure MeOH as extraction solvent was compared to those with MeOH + 0.4 M NH_4_Ac. The results show that addition of NH_4_Ac led to a significantly higher extraction yield of 7.8 μg F/g compared to 4.1 μg F/g with pure MeOH which exhibits 47% less organic fluorine (Figure 3 and Table S12). This underlines the importance of ammonia buffer addition for extractions of AFFF-contaminated soils dominated by cationic and zwitterionic PFAS.^45,46^

To determine the extraction recoveries for the applied extraction method (MeOH + 0.4 mM NH_4_Ac), a spike experiment on a standard soil was conducted with a PFAS standard mix (Table S1). Consecutive extraction with MeOH + 0.4 mM NH_4_Ac yielded extraction recoveries between 22.5 and 176.8% for cationic and zwitterionic PFAS. In total, only 8 out of 25 compounds showed recoveries between 70 and 130% (Table S13). However, most compounds with available IS were subject to a considerable ME, indicating that the extraction recoveries for other compounds might also be biased. This underlines the significance of MEs in (semi)quantification methods and the importance of correcting for it, for instance, by a matrix-matched approach.

### PFAS Concentrations in Deeper Soil Levels

**Figure 4:**
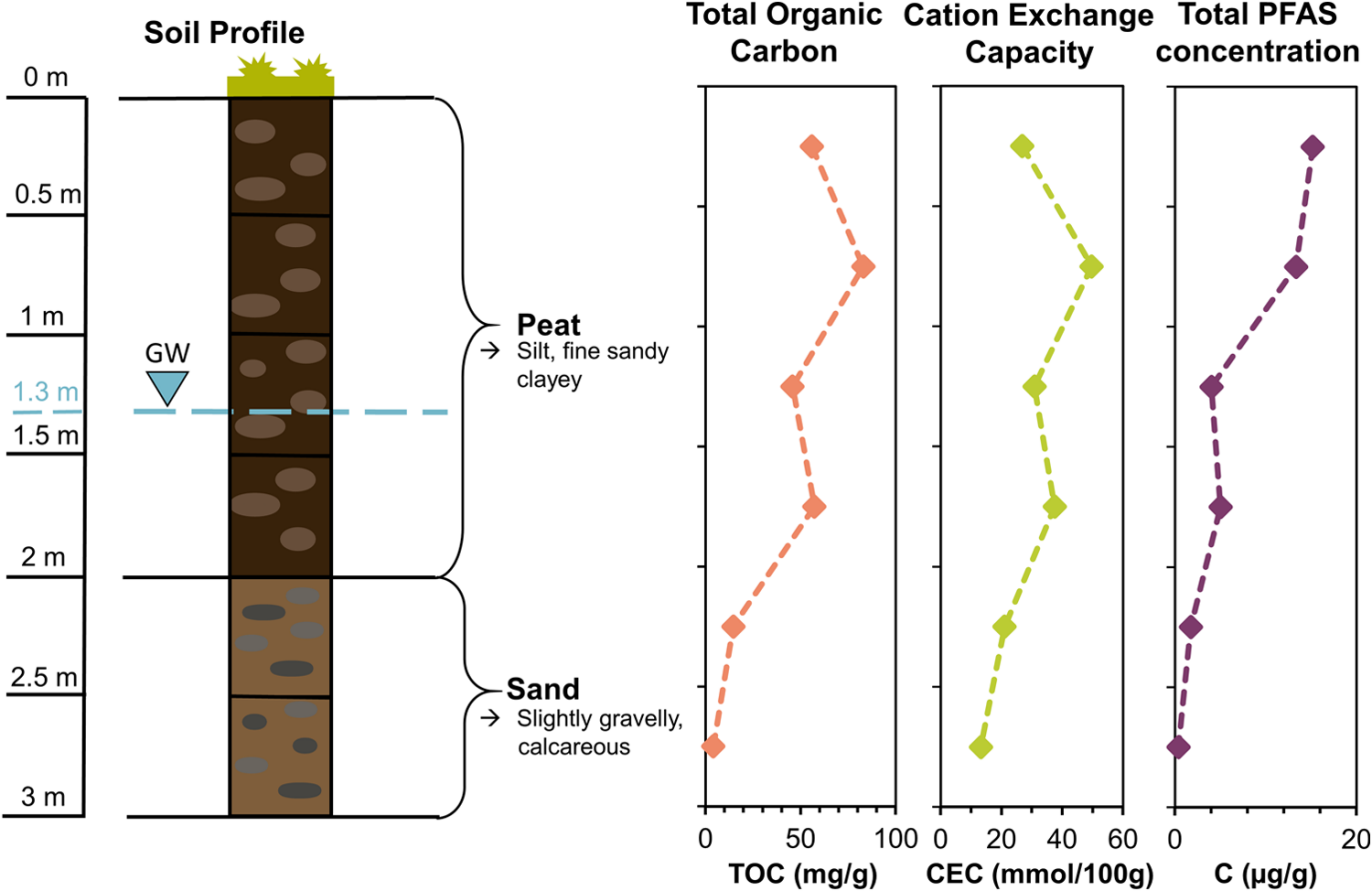
Soil profile for the six layers of *S1*(*L1–L6*) with groundwater level (GW), and trends for total organic carbon, cation exchange capacity, and total PFAS concentrations of all quantified and semiquantified PFAS from depths of 0–3 m.

**Figure 5:**
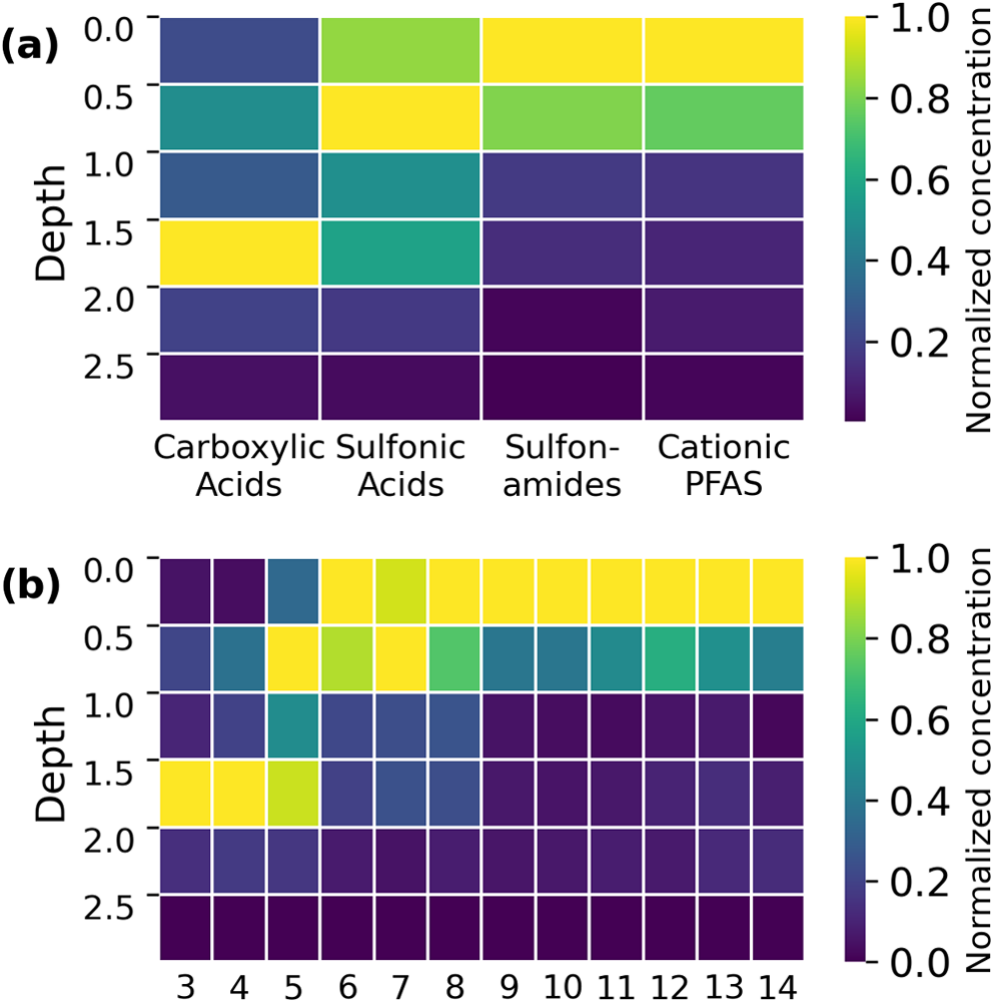
Depth distribution of the sum concentrations segmented by (a) ionization class and (b) chain length, depicted for a normalized scale based on the maximum concentration within an ionization class or chain length, respectively.

Generally, the sum of quantified and semiquantified PFAS showed a decreasing concentration trend with depth. Although in the upper two layers 15.19 μg/g (*S1L1*, 0–0.5 m) and 13.34 μg/g (*S1L2*, 0.5–1 m) of PFAS are still found, the sum concentrations in the four bottom layers are only 4.03 μg/g (*S1L3*, 1–1.5 m), 5.02 μg/g (*S1L4*, 1.5–2 m), 1.75 μg/g (*S1L5*, 2–2.5 m), and 0.39 μg/g (*S1L6*, 2.5–3 m) (Figure 4, individual concentrations in Table S11). This means that the majority of the contamination is still present in the top 1 m soil layer (*S1L1* and *S1L2*). This is consistent with findings from other field studies^43,47,48^ and may be caused by known retardation mechanisms like adsorption to solids and to the air–water interface. In the top first meter of soil, cationic/zwitterionic PFAS and sulfonamides constitute the largest proportion (Figures 5a and S6). Interestingly, some AFFF constituents (1.68 μg/g 5:2:1 FTB, 0.29 μg/g 5:3 FTB, 0.30 μg/g 6:2 fluorotelomer sulfonamide propyl methylamine (FTSAm-Pr-MeAm), and 0.91 μg/g 6:2 fluorotelomer sulfonamide propyl dimethylamine (FTSAm-Pr-DiMeAm)) and the potential AFFF degradation product 6:2 fluorotelomer sulfonic acid (FTSA, 0.85 μg/g) occur in the second top layer (*S1L2*, 0.5 m) at much higher concentrations than in the topsoil. This behavior may be explained by increased CEC and TOC values in *S1L2* (Figure 4) and therefore increased electrostatic interaction with cationic and zwitterionic PFAS, also observed in other studies.^49,50^ However, sorption of zwitter- and cationic PFAS was shown to be complex and not be primarily determined by soil characteristics^51^ and can be significantly affected by the presence of further foam constituents in AFFF formulations^52−54^ and fluorophilic interactions among the PFAS themselves during the input event and later periods.^55^

The anionic, rather mobile final TPs PFPeA, PFHxA, perfluoroheptanoic acid (PFHpA), and perfluorohexanesulfonic acid (PFHxS) show high concentrations in the saturated zone (Table S14), peaking in the fourth layer (*S1L4*, 1.5–2 m, Figures 5 and S6). This may represent a snapshot of the processes that are composed of in situ transformation of precursors in the two topsoil layers and the retarded transport of the TPs with the seepage water. It is known that sorption of anionic per- and polyfluoroalkyl acids (PFAAs) decreases with decreasing chain length and soil TOC^56^ and the more rapid release of PFAAs from AFFF-impacted soils was also described in the literature.^57^

### Strengths and Limitations of the Semiquantification Approach

The presented semiquantification approach demonstrates several notable strengths that highlight its effectiveness and reliability. Key features are the classification into specific ionization classes based on the known structures of the compounds and the inclusion of matrix-matched calibration, which both significantly improved the accuracy of the semiquantification.

Limitations of the approach arise from the fact that the structures of the analytes must be known for the classification and calibration and that the calibration must be adapted for each sample matrix. The method is also restricted to well-defined substance classes, and its applicability is dependent on the availability of suitable analytical standards for each ionization class. Substances that fall outside these classes pose challenges for this method due to the lack of matching standards. The accuracy could be improved by refining more groups, provided that suitable reference standards are available.

Given these constraints, predictive methods for ionization efficiency present an opportunity for future development. By integration of such methods, the limitations related to standard availability could be mitigated, expanding the applicability of semiquantification techniques.

Overall, the results emphasize the necessity of applying semiquantification approaches in nontarget screening studies. This approach has proven to be essential for characterizing PFAS contamination more comprehensively, providing a robust basis for remediation and risk assessment efforts.

## Supporting Information Available

Chemicals; sampling site; LC-MS parameters; details to all (semi)quantified PFAS; semiquantification method (standards for individual ACCs, scheme); extractable organofluorine method; method validation (ACC of all anionic PFAS, MEs); concentrations of individual PFAS; extractable organofluorine values; extraction recovery; and depth distribution (PDF)

## Abbreviations

AAQ: absolute accuracy quotient
ACC: average calibration curve
AFFF: aqueous film-forming foams
CE: collision energy
CEC: cation exchange capacity
CIC: combustion ion chromatography
ddMS_2_: data-dependent MS_2_ mode
EOF: extractable organofluorine
ESI: electrospray ionization
FTB: fluorotelomer betaine
FTSA: fluorotelomer sulfonic acid
FTSAm: fluorotelomer sulfonamide
FTSAm-Pr-B: fluorotelomer sulfonamide propyl betaine
FTSAm-Pr-DiMeAm: fluorotelomer sulfonamide propyl dimethylamine
FTSAm-Pr-MeAm: fluorotelomer sulfonamide propyl methylamine
FTSO-(2′)OHPr-TriMeAm: fluorotelomer sulfoxide propanol trimethylamine
FTSy-(2′)OHPr-TriMeAm: fluorotelomer sulfone propanol trimethylamine
HPLC: high-performance liquid chromatography
HRMS: high-resolution mass spectrometry
IC: ion chromatography
IS: mass-labeled internal standard
LC: liquid chromatography
LOQ: limit of quantification
m/C: mass per carbon number
M8PFOS: perfluoro-[^13^C_8_]octanesulfonic acid
M9PFNA: perfluoro-n-[^13^C_9_]nonanoic acid
MD/C: mass defect per carbon number
ME: matrix effect
MeOH: methanol
MP-AES: microwave plasma–atomic emission spectrometer
NH_4_Ac: ammonium acetate
NTS: nontarget screening
PFAA: per- and polyfluoroalkyl acid
PFAS: per- and polyfluoroalkyl substances
PFCA: perfluoroalkyl carboxylic acid
PFHpA: perfluoroheptanoic acid
PFHxA: perfluorohexanoic acid
PFHxS: perfluorohexanesulfonic acid
PFNA: perfluorononanoic acid
PFOA: perfluorooctanoic acid
PFOS: perfluorooctanesulfonic acid
PFPeA: perfluoropentanoic acid
PFSA: perfluoroalkyl sulfonic acid
PP: polypropylene
QC: quality control
QTOF: quadrupole time-of-flight
rcf: relative centrifugal forces
RF: response factor
RSD: relative standard deviation
SI: Supporting Information
TOC: total organic carbon
TP: transformation product
