# Supplementary material for: Implementation of Matrix-Matched Semiquantification of PFAS in AFFF-Contaminated Soil
Source: Environ Sci Technol. 2025 Apr 3;59(14):7338–47. doi: 10.1021/acs.est.4c14255 (PMC12004909; doi:10.1021/acs.est.4c14255)
Supplement: Supplementary file 1 — es4c14255_si_001.pdf [file es4c14255_si_001.pdf]

Supporting Information for

Implementation of matrix-matched semiquantification of PFAS in AFFF contaminated soil

Catharina Capitain[‡,⊥], Melanie Schüßler[‡,⊥], Boris Bugsel[⊥], Jonathan Zweigle[†], Christian Vogel[§], Peter Leube[§], Christian Zwiener[,⊥]*

[⊥]Environmental Analytical Chemistry, Department of Geosciences, University of Tübingen, Schnarrenbergstraße 94-96, 72076 Tübingen, Germany

[†]Department of Plant and Environmental Sciences, University of Copenhagen, Thorvaldsensvej 40, Frederiksberg 1871, Denmark

[§]Federal Institute for Materials Research and Testing, Division 4.4 – Thermochemical Residues Treatment and Resource Recovery, Unter den Eichen 87, 12205 Berlin, Germany

Number of pages: 24

Number of Tables: 14

Number of Figures: 6

Table of content

A. Chemicals 3
B. Sampling site 5
C. LC-MS parameters 5
D. Details to all (semi)quantified PFAS 6
E. Semiquantification method 12
F. EOF method – Combustion ion chromatography settings 13
G. Method validation 15
H. Concentrations of individual PFAS in topsoil 17
I. Concentrations of individual PFAS in all depths 18
J. Extractable organofluorine 21
K. Extraction recovery 22
L. Depth distribution 23
References 24

A. Chemicals

Table S1. Overview of all PFAS reference standards that were included in the PFAS standard mixture and where they were purchased. PFAS originated either from Wellington Laboratories, Guelph, Ontario, Canada (1), Toronto Research Chemicals, North York, Ontario, Canada (2), Dr. Ehrenstorfer, Augsburg, Bavaria, Germany (3) or were custom-synthesized in-house (4).

Acronym	Used standard / purchased chemical	Origin
**PFCAs**		
PFBA	Perfluorobutanoic acid / perfluoro-n-butanoic acid	(1)
PFPeA	Perfluoropentanoic acid / perfluoro-n-pentanoic acid	(1)
PFHxA	Perfluorohexanoic acid / perfluoro-n-hexanoic acid	(1)
PFHpA	Perfluoroheptanoic acid / perfluoro-n-heptanoic acid	(1)
PFOA	Perfluorooctanoic acid / perfluoro-n-octanoic acid	(1)
PFNA	Perfluorononanic acid / perfluoro-n-nonanic acid	(1)
PFDA	Perfluorodecanoic acid / perfluoro-n-decanoic acid	(1)
PFUnDA	Perfluoroundecanoic acid / perfluoro-n-undecanoic acid	(1)
PFDoDA	Perfluorododecanoic acid / perfluoro-n-dodecanoic acid	(1)
PFTriDA	Perfluorotridecanoic acid / perfluoro-n-tridecanoic acid	(1)
PFTeDA	Perfluorotetradecanoic acid / perfluoro-n-tetradecanoic acid	(1)
PFHxDA	Perfluorohexadecanoic acid / perfluoro-n-hexadecanoic acid	(1)
PFODA	Perfluorooctadecanoic acid / perfluoro-n-octadecanoic acid	(1)
**PFSAs**		
PFBS	Perfluorobutanesulfonic acid / potassium perfluoro-1-butanesulfonate	(1)
PFPeS	Perfluoropentanesulfonic acid / sodium perfluoro-1-pentanesulfonate	(1)
PFHxS	Perfluorohexanesulfonic acid / sodium perfluoro-1-hexanesulfonate	(1)
PFHpS	Perfluoroheptanesulfonic acid / sodium perfluoro-1-heptanesulfonate	(1)
PFOS	Perfluorooctanesulfonic acid / sodium perfluoro-1-octanesulfonate	(1)
PFNS	Perfluorononanesulfonic acid / sodium perfluoro-1-nonanesulfonate	(1)
PFDS	Perfluorodecanesulfonic acid / sodium perfluoro-1-decanesulfonate	(1)
PFDoDS	Perfluorododecanesulfonic acid / sodium perfluoro-1-dodecanesulfonate	(1)
**PFPAs**		
PFOPA	Perfluorooctylphosphonic acid	(1)
PFDPA	Perfluorodecylphosphonic acid	(1)
**PAPs**		
6:2/6:2 diPAP	6:2/6:2 phosphoric acid diester / Bis[2-(perfluorohexyl)ethyl]phosphate	(2)
8:2/8:2 diPAP	6:2/6:2 polyfluoroalkyl phosphoric acid diester / Sodium bis (1H, 1H, 2H, 2H-perfluorodecyl) phosphate	(1)
6:2 PAP	6:2 polyfluoroalkyl phosphoric ester / Mono[2-(perfluorohexyl)ethyl] phosphate	(2)
8:2 PAP	6:2 polyfluoroalkyl phosphoric ester / Sodium 1H, 1H, 2H, 2H-perfluorodecyl phosphate	(1)
**PFPiAs**		
C6/C6 PFPiA	C6/C6 Perfluoroalkyl phosphinic acid	(2)
**PASF-based PFAS**		
PFHxSAm	Perfluorohexane sulfonamide	(3)
PFOSAm	Perfluorooctane sulfonamide	(1)
PFOSAm-*N*-Et-*N*-EtA	Perfluorooctane sulfonamide *N*-ethyl *N*-ethanoic acid	(1)
SamPAP	Perfluorooctane sulfonamide ethanol-based phosphate diester /Sodium-2-(N-ethylperfluorooctane-1-sulfonamido) ethyl phosphate	(1)
diSAmPAP	Perfluorooctane sulfonamide ethanol-based phosphate diester/ Sodium bis[2-(N-ethylperfluorooctane-1-sulfonamido) ethyl] phosphate	(1)
**FTCAs**		
6:2 FTCA	6:2 fluorotelomer carboxylic acid / 2-Perfluorohexyl ethanoic acid (6:2)	(1)

8:2 FTCA	8:2 fluorotelomer carboxylic acid / 2-Perfluorooctyl ethanoic acid (6:2)	(1)
5:3 FTCA	5:3 fluorotelomer carboxylic acid/ 3-Perfluoropentyl propanoic acid	(1)
7:3 FTCA	7:3 fluorotelomer carboxylic acid/ 3-Perfluoroheptyl propanoic acid	(1)
**FTUCAs**		
6:2 FTUCA	6:2 fluorotelomer unsaturated carboxylic acid/ 2H-Perfluoro-2-octenoic acid (6:2)	(1)
8:2 FTUCA	8:2 fluorotelomer unsaturated carboxylic acid/ 2H-Perfluoro-2-decenoic acid (8:2)	(1)
**FTSAs**		
6:2 FTSA	6:2 fluorotelomer sulfonic acid / Sodium 1H, 1H,2H,2H-perfluorooctanesulfonate	(1)
8:2 FTSA	8:2 fluorotelomer sulfonic acid / Sodium 1H, 1H,2H,2H-perfluorodecanesulfonate	(1)
**FTMAPs**		
6:2 FTMAP	6:2 Fluorotelomer mercapto alkyl phosphate	(4)
**PFECAs**		
HFPO-Da	2,3,3,3-Tetrafluoro-2-(1,1,2,2,3,3,3-heptafluoropropoxy) propanoic acid	(1)
ADONA	Sodium dodecafluoro-3H-4,8-dioxanonanoate	(1)
**PFESAs**		
9Cl-PF3ONS	Potassium 9-chlorhexadecafluoro-3-oxanonane-1-sulfonate	(1)
11Cl-PF3OUdS	Potassium 11-chloroeicosafluoro-3-oxaundecane-1-sulfonate	(1)
**AFFF-substances**		
6:2 FTSAm-Pr-DiMeNO	6:2 fluorotelomer sulfonamide propyl methylamineoxide / Capstone product A	(3)
6:2 FTSAm-Pr-B	6:2 fluorotelomer sulfonamide propyl betaine / Capstone product B	(3)
PFHxSAm-Pr-DiMeAm	N-[3(dimethylamino)propyl] perfluoro-1-hexanesulfonamide	(3)
5:3 FTB	5:3 Fluorotelomer Betaine / 2-[4,4,5,5,6,6,7,7,8,8,8-Undecafluorooctyl) dimethylammonio] acetate	(1)
5:1:2 FTB	5:1:2 Fluorotelomer Betaine / 2-[(3,4,4,5,5,6,6,7,7,8,8,8-Dodecafluorooctyl) dimethylammonio] acetate	(1)

Table S2. Internal standards that were used for quantification of the matrix effect in the samples. Internal standards were acquired from Wellington laboratories.

Native PFAS	Mass labeled internal standard
PFPeA	M5PFPeA
PFHxA	M5PFHxA
PFHpA	M4PFHpA
PFOA	M8PFOA
PFNA	M9PFNA
PFDA	M6PFDA
PFUnDA	M7PFUnDA
PFDoDA	MPFDoDA
PFTeDA	M2PFTeDA
PFBS	M3PFBS
PFHxS	M3PFHxS
PFOS	M8PFOS

B. Sampling site

Sampling Site (Soil)	Coordinates
S1-1	49°17'49.2396"N<br>8°33'40.8816"E
S1-2	49°17'49.2036"N<br>8°33'40.9284"E
S1-3	49°17'49.1712"N<br>8°33'40.8816"E
S1-4	49°17'49.2072"N<br>8°33'40.8312"E

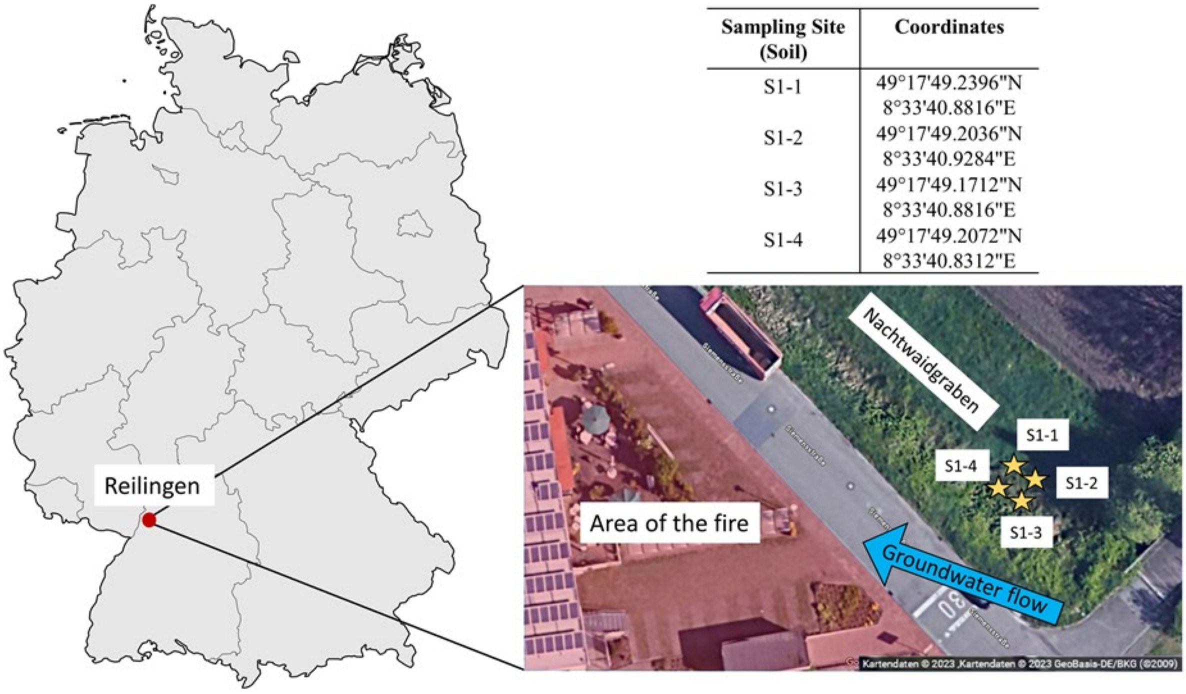

Figure S1. Overview of the sampling site 'Nachtwaidgraben' in Reilingen, Baden Württemberg and coordinates of the respective sampling points. Yellow stars represent the soil sampling points. The screenshot of the field site was taken from Google Maps (15.12.2023). Groundwater flow was determined by Arcadis. Source of map: Wikimedia Commons [1].

C. LC-MS parameters

Table S3. Elution gradient of the applied HPLC-QTOF Method with eluent A (95/5 H_2O/MeOH + 2 mM NH_4Ac) and eluent B (95/5 MeOH/H_2O + 2mM NH_4Ac).

Time (min)	Eluent A (%)	Eluent B (%)
0.0	85	15
2.0	30	70
5.0	10	90
10.0	0	100
15.0	0	100
15.1	85	15
22.0	85	15

D. Details to all (semi)quantified PFAS

Table 4: Acronyms and full names of subclasses/compounds with an example structure, formula, confidence level, ion species and theoretical m/z. Substance classes are organized according to the four ionizing groups of the ACCs.

Subclass	Example structure	Acronym	Neutral formula [M]	Confidence Level	Ion	Theoretical m/z
**Ionization class: Carboxylic Acids**						
PFCA *Perfluoro carboxylic acid*	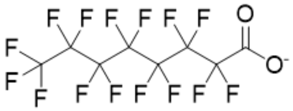	PFBA	C4HF7O2	1	[M-H]-	212.9792
		PFPeA	C5HF9O2	1	[M-H]-	262.9760
		PFHxA	C6HF11O2	1	[M-H]-	312.9728
		PFHpA	C7HF13O2	1	[M-H]-	362.9696
		PFOA	C8HF15O2	1	[M-H]-	412.9664
		PFNA	C9HF17O2	1	[M-H]-	462.9632
		PFDA	C10HF19O2	1	[M-H]-	512.9600
		PFUnDA	C11HF21O2	1	[M-H]-	562.9568
		PFDoDA	C12HF23O2	1	[M-H]-	612.9537
PFASAm-EtA *Perfluoroalkane sulfonamide ethanoic acid*	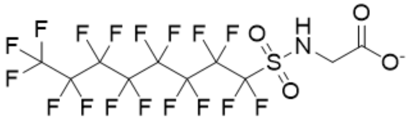	PFHxSAm-EtA	C8H4F13NO4S	2	[M-H]-	455.9581
		PFOSAm-EtA	C10H4F17NO4S	2	[M-H]-	555.9517
PFASAm-Pr-B *Perfluoroalkane sulfonamide propyl betaine*	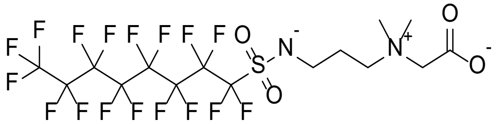	PFBSAm-Pr-B	C11H15F9N2O4S	2	[M+H]+	443.0682
		PFHxSAm-Pr-B	C13H15F13N2O4S	2	[M+H]+	543.0618
					[M-H]-	541.0472
		PFOSAm-Pr-B	C15H15F17N2O4S	2	[M+H]+	643.0554
					[M-H]-	641.0408
n:3 FTCA *n:3 fluorotelomer carboxylic acid*	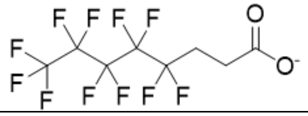	5:3 FTCA	C8H5F11O2	1	[M-H]-	341.0041
		7:3 FTCA	C10H5F15O2	1	[M-H]-	440.9977
n:2 FTSAm-Pr-B *n:2 fluorotelomer sulfonamide propyl betaine*	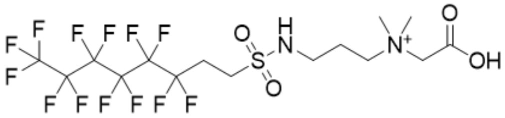	6:2 FTSAm-Pr-B	C15H19F13N2O4S	1	[M+H]+	571.0931
					[M-H]-	569.0785
		8:2 FTSAm-Pr-B	C17H19F17N2O4S	2	[M+H]+	671.0867

n:2 FTSAm-PrA<br>*n:2 fluorotelomer sulfonamide propanoic acid*	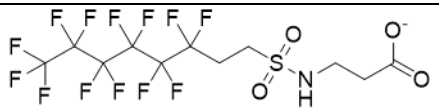	6:2 FTSAm-PrA	C11H10F13NO4S	2	[M-H]-	498.0050
n:2 FTSAm-*N*-Me-*N*-PrA<br>*n:2 fluorotelomer sulfonamide N-methyl N-propanoic acid*	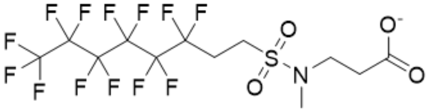	6:2 FTSAm-*N*-Me-*N*-PrA	C12H12F13NO4S	2	[M+H]+	514.0352
					[M-H]-	512.0207
n:2 FTSy-PrA<br>*n:2 fluorotelomer sulfone propanoic acid*	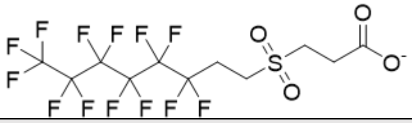	6:2 FTSy-PrA	C11H9F13O4S	2	[M-H]-	482.9941
		8:2 FTSy-PrA	C13H9F17O4S	2	[M-H]-	582.9877
**Ionization class: Sulfonic Acids**						
PFSA<br>*Perfluoro sulfonic acid*	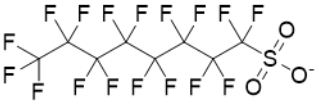	PFPrS	C3HF7O3S	2	[M-H]-	248.9462
		PFBS	C4HF9O3S	1	[M-H]-	298.9430
		PFPeS	C5HF11O3S	1	[M-H]-	348.9398
		PFHxS	C6HF13O3S	1	[M-H]-	398.9366
		PFHpS	C7HF15O3S	1	[M-H]-	448.9334
		PFOS	C8HF17O3S	1	[M-H]-	498.9302
		PFNS	C9HF19O3S	1	[M-H]-	548.9270
U-PFSA<br>*Unsaturated perfluoro sulfonic acid*	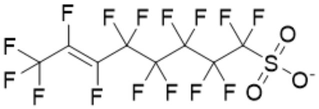	U-PFHxS	C6HF11O3S	2	[M-H]-	360.9398
		U-PFHpS	C7HF13O3S	2	[M-H]-	410.9366
		U-PFOS	C8HF15O3S	2	[M-H]-	460.9334
		U-PFNS	C9HF17O3S	2	[M-H]-	510.9302
		U-PFDS	C10HF19O3S	2	[M-H]-	560.9270
		U-PFUnDS	C11HF21O3S	2	[M-H]-	610.9238
		U-PFDoDS	C12HF23O3S	2	[M-H]-	660.9206
		U-PFTriDS	C13HF25O3S	2	[M-H]-	710.9174
		U-PFTeDS	C14HF27O3S	2	[M-H]-	760.9143
PFASyA<br>*Perfluoro sulfinic acid*	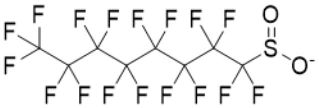	PFOSyA	C8HF17O2S	2	[M-H]-	482.9353
SF_5-PFSA<br>*Pentafluorosulfanyl perfluoro sulfonic acid*	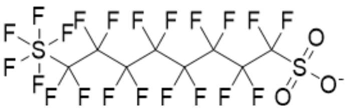	SF_5-PFHpS	C7HF19O3S2	2	[M-H]-	556.8991
		SF_5-PFOS	C8HF21O3S2	2	[M-H]-	606.8959
		SF_5-PFNS	C9HF23O3S2	2	[M-H]-	656.8927
		SF_5-PFDS	C10HF25O3S2	2	[M-H]-	706.8895
		SF_5-U-PFOS	C8HF19O3S2	2	[M-H]-	568.8991

SF_5-U-PFSA<br>*Pentafluorosulfanyl unsaturated perfluoro sulfonic acid*	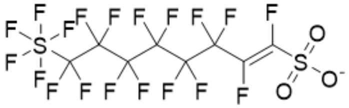	SF_5-U-PFNS	C9HF21O3S2	2	[M-H]-	618.8959
Cl-PFSA<br>*Chloro substituted perfluoro sulfonic acid*	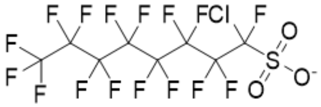	Cl-PFOS	C8HClF16O3S	2	[M-H]-	514.9007
U-E-PFSA/K-PFSA<br>*Unsaturated ether perfluoro sulfonic acid/Ketone perfluoro sulfonic acid*	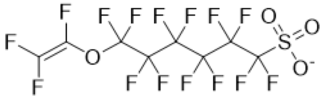	U-E-PFHpS/K-PFHxS	C6HF11O4S	3	[M-H]-	376.9347
	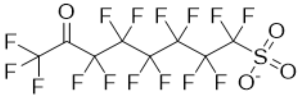	U-E-PFOS/K-PFHpS	C7HF13O4S	3	[M-H]-	426.9315
		U-E-PFNS/K-PFOS	C8HF15O4S	3	[M-H]-	476.9283
		U-E-PFTriDS/K-PFDoDS	C12HF23O4S	3	[M-H]-	676.9156
H-PFSA<br>*H-substituted perfluoro sulfonic acid*	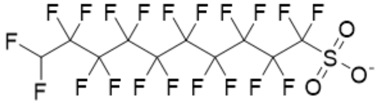	H-PFOS	C8H2F16O3S	2	[M-H]-	480.9396
		H-PFDS	C10H2F20O3S	3	[M-H]-	580.9333
PFASAm-PrSA<br>*Perfluoroalkane sulfonamide propyl sulfonic acid*	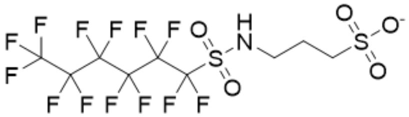	PFHxSAm-PrSA	C9H8F13NO5S2	2	[M-H]-	519.9564
PFASAm-*N*-PrSA-*N*-Pr-DiMeAm<br>*Perfluoroalkane sulfonamide N-propyl sulfonic acid N-propyl dimethylamine*	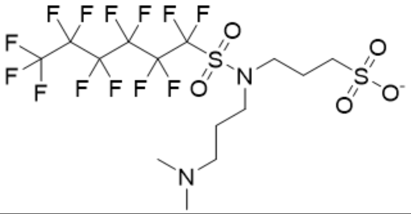	PFHxSAm-*N*-PrSA-*N*-Pr-DiMeAm	C14H19F13N2O5S2	3	[M+H]+	607.0601
					[M-H]-	605.0455
n:2 FTSA<br>*n:2 fluorotelomer sulfonic acid*	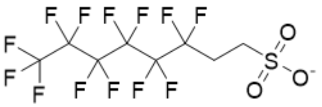	6:2 FTSA	C8H5F13O3S	1	[M-H]-	426.9679
		8:2 FTSA	C10H5F17O3S	1	[M-H]-	526.9615
		10:2 FTSA	C12H5F21O3S	2	[M-H]-	626.9551
		12:2 FTSA	C14H5F25O3S	2	[M-H]-	726.9487
		14:2 FTSA	C16H5F29O3S	2	[M-H]-	826.9424
OH-n:2 FTSA<br>*Hydroxy-n:2 fluorotelomer sulfonic acid*	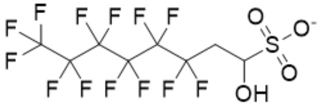	OH-6:2 FTSA	C8H5F13O4S	2	[M-H]-	442.9628
		OH-8:2 FTSA	C10H5F17O4S	2	[M-H]-	542.9564
K-n:2 FTSA<br>*Ketone-n:2 fluorotelomer sulfonic acid*	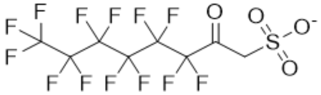	K-6:2 FTSA	C8F13H3SO4	2	[M-H]-	440.9472
		K-8:2 FTSA	C10F17H3SO4	2	[M-H]-	540.9408

U-n:2 FTSA<br>*Unsaturated n:2 fluorotelomer sulfonic acid*	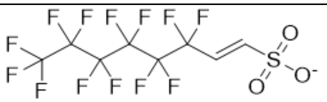	U-6:2 FTSA	C8H3F13O3S	2	[M-H]-	424.9523
		U-8:2 FTSA	C10H3F17O3S	3	[M-H]-	524.9459
n:2 FTSO-Pr-Ad-(5’,5’)DiMeEtSA<br>*n:2 fluorotelomer sulfoxide propyl amide dimethylethyl sulfonic acid*	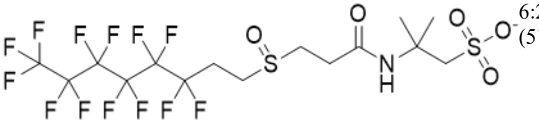	6:2 FTSO-Pr-Ad-(5’,5’)DiMeEtSA	C15H18F13NO5S2	2	[M-H]-	602.0346
n:2 FTSy-Pr-Ad-(5’,5’)DiMeEtSA<br>*n:2 fluorotelomer sulfone propyl amide dimethylethyl sulfonic acid*	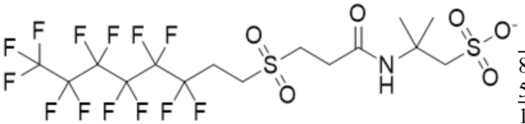	6:2 FTSy-Pr-Ad-(5’,5’)DiMeEtSA	C15H18F13NO6S2	2	[M-H]-	618.0295
		8:2 FTSy-Pr-Ad-(5’, 5’)DiMeEtSA	C17H18F17NO6S2	2	[M-H]-	718.0231
		10:2 FTSy-Pr-Ad-(5’,5’)DiMeEtSA	C19H18F21NO6S2	2	[M-H]-	818.0167
		12:2 FTSy-Pr-Ad-(5’,5’)DiMeEtSA	C21H18F25NO6S2	2	[M-H]-	918.0104
**Ionization class: Sulfonamides**						
PFASAm<br>*Perfluoroalkane sulfonamide*	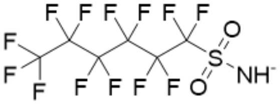	PFBSAm	C4H2F9NO2S	2	[M-H]-	297.9590
		PFPeSAm	C5H2F11NO2S	2	[M-H]-	347.9558
		PFHxSAm	C6H2F13NO2S	1	[M-H]-	397.9526
		PFHpSAm	C7H2F15NO2S	2	[M-H]-	447.9494
		PFOSAm	C8H2F17NO2S	1	[M-H]-	497.9462
PFASAm-Me<br>*Perfluoroalkane sulfonamide methyl*	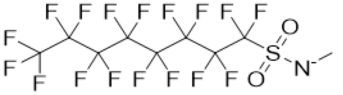	PFBSAm-Me	C5H4F9NO2S	3	[M-H]-	311.9746
		PFOSAm-Me	C9H4F17NO2S	3	[M-H]-	511.9619
n:2 FTSAm<br>*n:2 fluorotelomer sulfonamide*	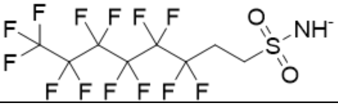	6:2 FTSAm	C8H6F13NO2S	2	[M-H]-	425.9839
		8:2 FTSAm	C10H6F17NO2S	2	[M-H]-	525.9775
		10:2 FTSAm	C12H6F21NO2S	2	[M-H]-	625.9711
n:2/m:2 FTSAm dimer<br>*n:2/m:2 fluorotelomer sulfonamide dimer*	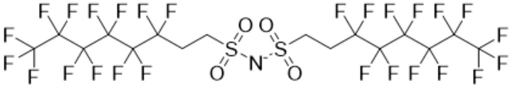	6:2/6:2 FTSAm dimer	C16H9F26NO4S2	2	[M-H]-	835.9485
		6:2/8:2 FTSAm dimer	C18H9F30NO4S2	2	[M-H]-	935.9421

**Ionization class: Cationic PFAS**						
PFASAm-Pr-DiMeAm *Perfluoroalkane sulfonamide propyl dimethylamine*	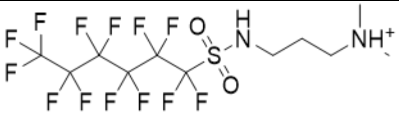	PFHxSAm-Pr-DiMeAm	C11H13F13N2O2S	1	[M+H]+	485.0563
					[M-H]-	483.0417
		PFOSAm-Pr-DiMeAm	C13H13F17N2O2S	2	[M+H]+	585.0499
					[M-H]-	583.0353
PFASAm-Pr-TriMeAm *Perfluoroalkane sulfonamide propyl trimethylamine*	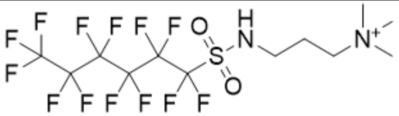	PFHxSAm-Pr-TriMeAm	C12H15F13N2O2S	3	[M+H]+	499.0719
		PFOSAm-Pr-TriMeAm	C14H15F17N2O2S	2	[M+H]+	599.0656
n:1:2 FTB *n:1:2 fluorotelomer betaine*	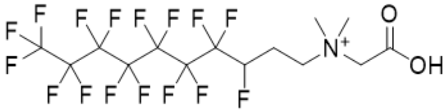	5:1:2 FTB	C12H13F12NO2	1	[M+H]+	432.0827
		7:1:2 FTB	C14H13F16NO2	2	[M+H]+	532.0764
		9:1:2 FTB	C16H13F20NO2	2	[M+H]+	632.0700
		11:1:2 FTB	C18H13F24NO2	2	[M+H]+	732.0636
		13:1:2 FTB	C20H13F28NO2	2	[M+H]+	832.0572
n:1:3 FTB *n:1:3 fluorotelomer betaine*	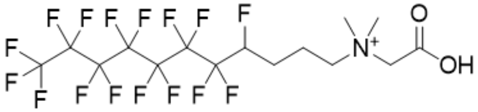	5:1:3 FTB	C13H15F12NO2	3	[M+H]+	446.0984
		7:1:3 FTB	C15H15F16NO2	3	[M+H]+	546.0920
		9:1:3 FTB	C17H15F20NO2	3	[M+H]+	646.0856
n:2 FTB *n:2 fluorotelomer betaine*	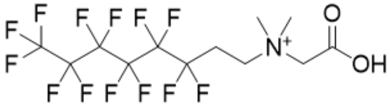	6:2 FTB	C12H12F13NO2	2	[M+H]+	450.0733
		8:2 FTB	C14H12F17NO2	2	[M+H]+	550.0669
		10:2 FTB	C16H12F21NO2	2	[M+H]+	650.0605
n:3 FTB *n:3 fluorotelomer betaine*	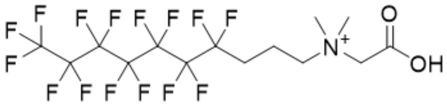	5:3 FTB	C12H14F11NO2	1	[M+H]+	414.0922
		7:3 FTB	C14H14F15NO2	2	[M+H]+	514.0858
		9:3 FTB	C16H14F19NO2	2	[M+H]+	614.0794
		11:3 FTB	C18H14F23NO2	2	[M+H]+	714.0730
		13:3 FTB	C20H14F27NO2	2	[M+H]+	814.0666
n:4 FTB *n:4 fluorotelomer betaine*	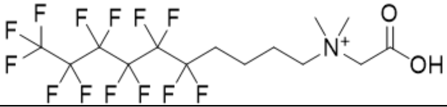	4:4 FTB	C12H16F9NO2	2	[M+H]+	378.1110
		6:4 FTB	C14H16F13NO2	2	[M+H]+	478.1046
		8:4 FTB	C16H16F17NO2	2	[M+H]+	578.0982
		10:4 FTB	C18H16F21NO2	2	[M+H]+	678.0918
n:2 FTSAm-Pr-DiMeAm *n:2 fluorotelomer sulfonamide propyl dimethylamine*	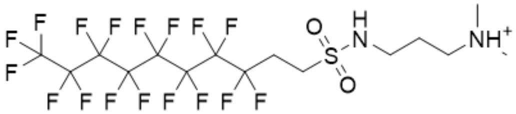	6:2 FTSAm-Pr-DiMeAm	C13H17F13N2O2S	2	[M+H]+	513.0876
					[M-H]-	511.0730
		8:2 FTSAm-Pr-DiMeAm	C15H17F17N2O2S	2	[M+H]+	613.0812

n:2 FTSAm-U-Pr-DiMeAm<br>*n:2 fluorotelomer sulfonamide unsaturated propyl dimethylamine*	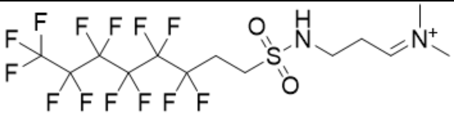	6:2 FTSAm-U-Pr-DiMeAm	C13H15F13N2O2S	2	[M+H]+	511.0719
n:2 FTSAm-Pr-DiMeNO<br>*n:2 fluorotelomer sulfonamide propyl dimethylamineoxide*	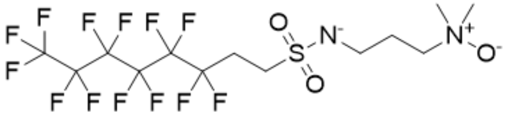	6:2 FTSAm-Pr-DiMeNO	C13H17F13N2O3S	1	[M+H]+	529.0825
					[M-H]-	527.0680
		8:2 FTSAm-Pr-DiMeNO	C15H17F17N2O3S	3	[M+H]+	629.0761
n:2 FTTh-(2')OHPr-TriMeAm<br>*n:2 fluorotelomer thio propanol trimethylamine*	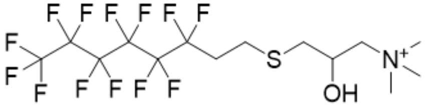	4:2 FTTh-(2')OHPr-TriMeAm	C12H18F9NOS	2	[M+H]+	396.1038
		6:2 FTTh-(2')OHPr-TriMeAm	C14H18F13NOS	2	[M+H]+	496.0974
		8:2 FTTh-(2')OHPr-TriMeAm	C16H18F17NOS	2	[M+H]+	596.0910
n:2 FTSO-(2')OHPr-TriMeAm<br>*n:2 fluorotelomer sulfoxide propanol trimethylamine*	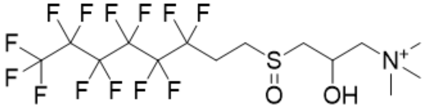	6:2 FTSO-(2')OHPr-TriMeAm	C14H18F13NO2S	2	[M+H]+	512.0923
		8:2 FTSO-(2')OHPr-TriMeAm	C16H18F17NO2S	2	[M+H]+	612.0860
n:2 FTSy-(2')OHPr-TriMeAm<br>*n:2 fluorotelomer sulfone propanol trimethylamine*	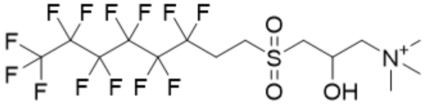	4:2 FTSy-(2')OHPr-TriMeAm	C12H18F9NO3S	2	[M+H]+	428.0936
		6:2 FTSy-(2')OHPr-TriMeAm	C14H18F13NO3S	2	[M+H]+	528.0873
		8:2 FTSy-(2')OHPr-TriMeAm	C16H18F17NO3S	2	[M+H]+	628.0809

E. Semiquantification method

Table S5. Analytical reference standards of the four average calibration curves for semiquantification.

**Carboxylic Acids**	**Sulfonic Acids**	**Sulfonamides**	**Cationic PFAS**
PFBA	PFBS	PFHxSAm	PFHxSAm-Pr-DiMeAm
PFPeA	PFPeS	PFOSAm	5:1:2 FTB
PFHxA	PFHxS	PFOSAm-*N*-Et-*N*-EtA	5:3 FTB
PFHpA	PFHpS	PFHxSAm-Pr-DiMeAm	6:2 FTSAm-Pr-B
PFOA	PFOS	6:2 FTSAm-Pr-B	6:2 FTSAm-Pr-DiMeNO
PFNA	PFNS	6:2 FTSAm-Pr-DiMeNO	
PFDA	PFDS		
PFUnDA	PFDoDS		
PFDoDA	6:2 FTSA		
PFTriDA	8:2 FTSA		
PFTeDA	9Cl-PF3ONS		
PFHxDA	11Cl-PF3OUdS		
PFODA			
PFOSAm-*N*-Et-*N*-EtA			
5:3 FTCA			
7:3 FTCA			
U-6:2 FTCA			
U-8:2 FTCA			
6:2 FTSAm-Pr-B			
PFDPA			
ADONA			

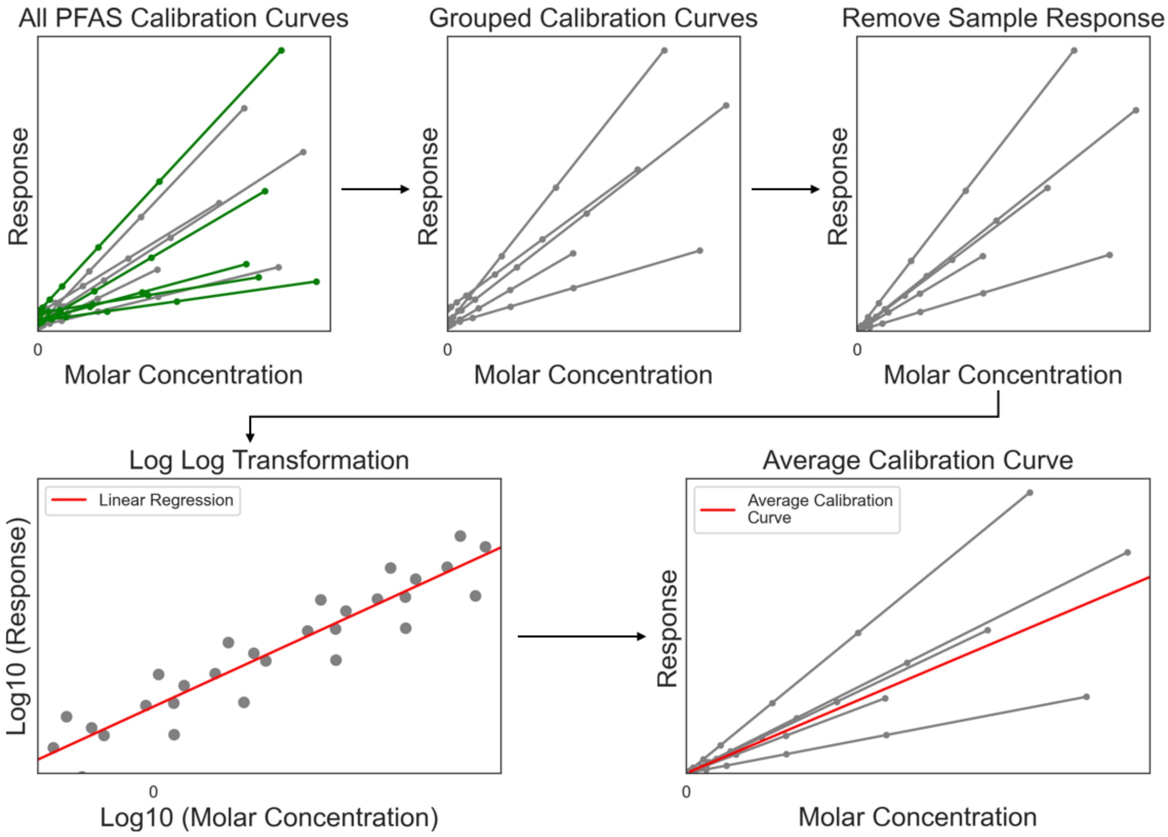

Figure S2. Scheme of semiquantification approach. The green lines represent calibration curves of authentic standards of other ionization classes.

F. EOF method – Combustion ion chromatography settings

Table S6. Combustion parameters.

**Combustion component**	
Combustion device	AQF-2100H, A1 Enviroscience, Mitsubishi Chemical Analytech Co., Ltd.
Operating temperature	1050 °C
Ar carrier gas flow	150 mL/min
Ar flow of water supply	100 mL/min
O_2 flow	300 mL/min
Absorption solution/internal standard	150 µM NH_3 solution + 2 mg/L $MeSO_3H$
Starting Absorption volume	3.5 mL
Final Absorption volume	~10.5 mL
Sample amount	250 µL
Water supply level	2 (about 0.1 mL/min)

Table S7. Ion chromatography parameters.

**Ion chromatography component**	
IC-device	ICS Integrion, Thermo Fisher Scientific
Detector	conductivity detector
Guard column	AG20 2x50mm guard column
Analytical column	Dionex IonPac AS20 2x250mm
Eluent	gradient KOH
Flow rate	0.25 mL/min
Run time	22 min
Column temperature	30 °C
Injection volume	100 µL
Suppressor	Dionex ADRS 600 (2mm)

Table S8. Boat program.

**Pos [mm]**	**Time [s]**	**Pos [mm]**	**Time [s]**	**Pos [mm]**	**Time [s]**	**Pos [mm]**	**Time [s]**	**End Time [s]**	**Cool Time [s]**	**Home Time [s]**	**Ar Time [s]**	**O2 Time [s]**
65	30	100	60	130	60	150	60	460	60	120	10	600

Table S9. IC eluent gradient program for all fluorine detection measurements.

**Time (min)**	**Eluent concentration (mM)**
0	Start, 1.0
0.1	1.0
0.2	2.0
1.0	2.0
10.0	5.0
10.5	5.0
11.0	80.0
14.0	80.0
14.5	1.0
22.0	stop run

G. Method validation

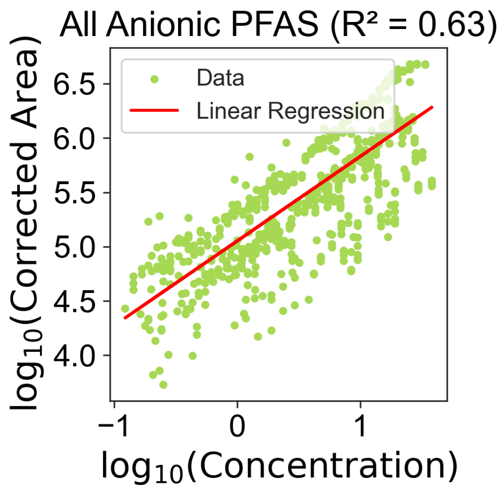

Figure S3. Data points of each standard of all anionic PFAS and average calibration curves displayed on a double logarithmic scale.

Cao, et al. [2] developed the ACC approach and re-estimated 50 different anionic and cationic PFAS, resulting in a median AAQ of 2.89 for the log-log regression approach. Pu, et al. [3] used, among others, bootstrap simulation to estimate population RF percentile values. Estimating all available PFAS standards together, similar to Cao, et al. [2], yielded with a median AAQ of 2.85, which could be decreased to 1.80 by using a suitable subset of the available standards based, chosen based on expert chemical intuition instead of all available surrogates. Lauria, et al. [4] reported mean AAQs of 1.90 when predicting the ionization efficiency of anionic PFAS using machine learning. Malm, et al. [5] evaluated the accuracy and performance variability of five quantification approaches based on surrogate standard quantification and predicted ionization efficiencies, reporting reprocessed mean AAQs ranging from 2.40 to 5.00.

Table S10. AAQs of different approaches compared.

Approach	AAQ	Reference
Average calibration curve (log-log regression)	Median: 2.89	Cao, et al. [2]
Bootstrap-sampled calibration values (“global” chemical surrogates)	Median: 2.85	Pu, et al. [3]
Bootstrap-sampled calibration values (“expert-selected” chemical surrogates)	Median: 1.80	Pu, et al. [3]
Machine learning predict the ionization efficiency	Mean: 1.90	Lauria, et al. [4]
Surrogate standard quantification (3)/Predicted ionization efficiencies (2)	Mean: 2.40-5.00	Malm, et al. [5]

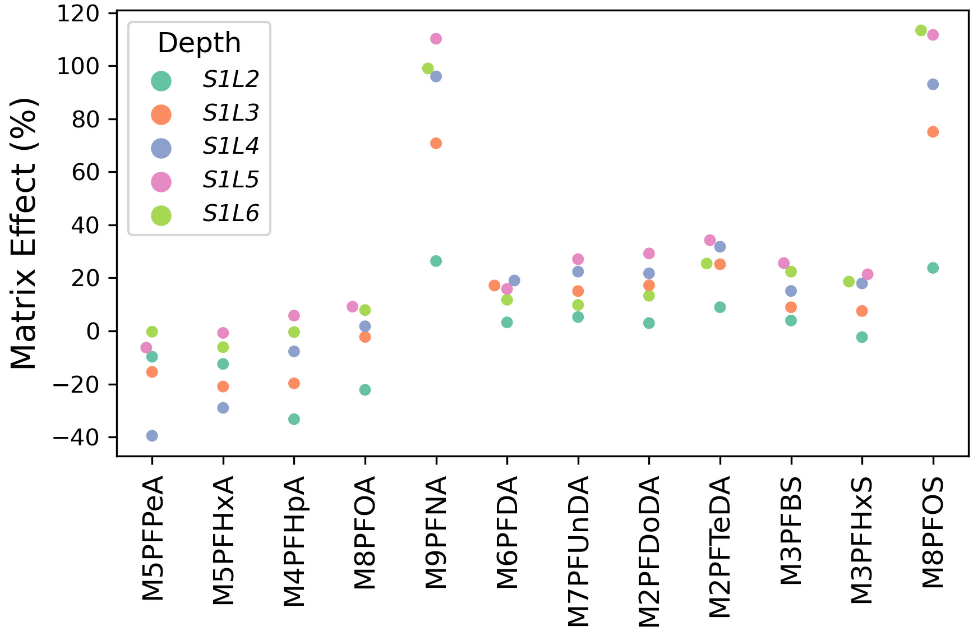

Figure S4. Matrix effects (MEs) of different isotopically labelled PFAS standards relative to the extract of the topsoil.

H. Concentrations of individual PFAS in topsoil

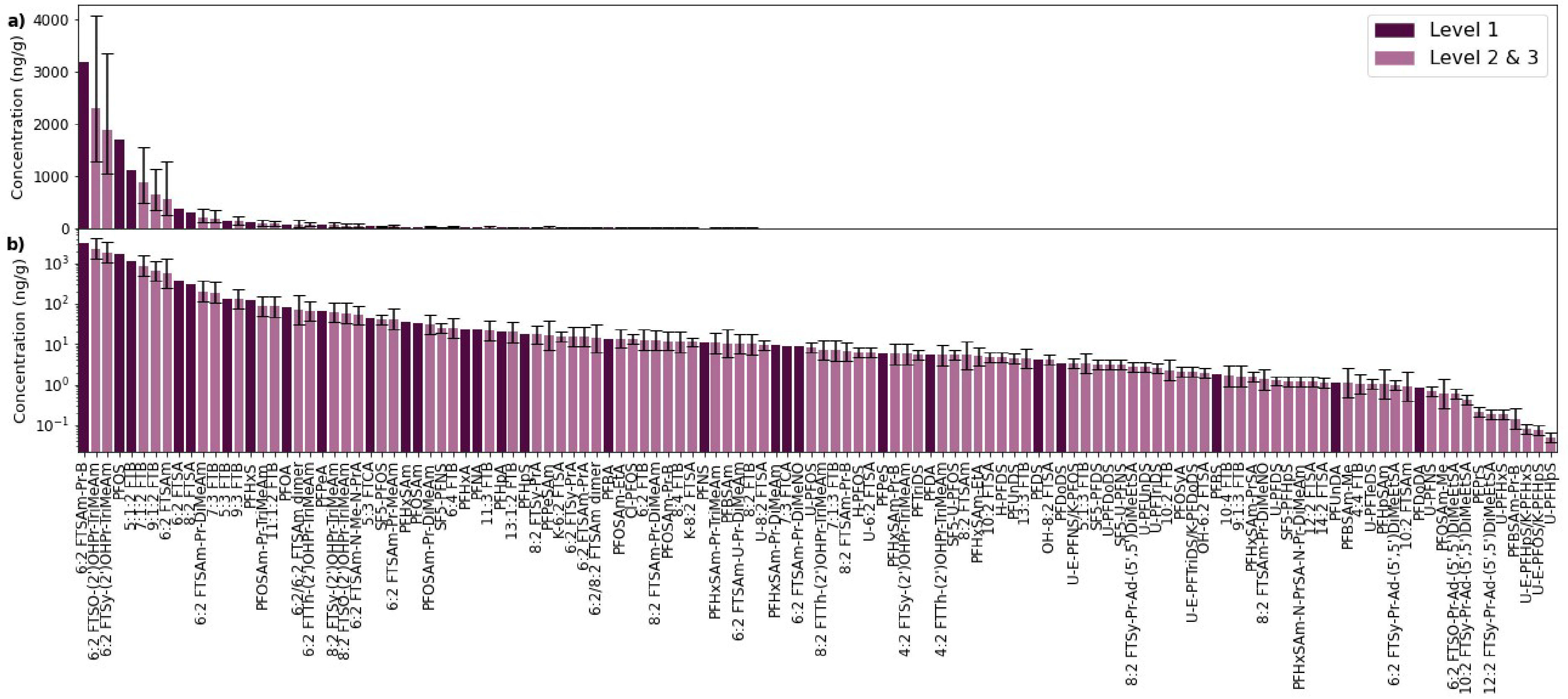

Figure S5. Concentration of quantified (dark purple) and semiquantified (light purple) PFAS in the extract of topsoil on site *S1L1* in µg/g displayed with a) linear scale and b) logarithmic scale. Error bars for semiquantified PFAS represent the median AAQ of the corresponding ionization class (compound names and structures in Table S4, concentrations in Table S12).

I. Concentrations of individual PFAS in all depths

Table S11. (Semi)quantified concentrations (ng/g) of all identified PFAS in different depths. Substances are organized in two groups, depending if they were quantified or semiquantified and ordered according to concentration in topsoil within each group.

	Acronym	C (ng/g) at 0-0.5 m	C (ng/g) at 0.5-1 m	C (ng/g) at 1-1.5 m	C (ng/g) at 1.5-2 m	C (ng/g) at 2-2.5 m	C (ng/g) at 2.5-3 m
Quantified	6:2 FTSAm-Pr-B	3168.96	1689.00	362.36	248.77	186.58	72.83
	PFOS	1696.75	1486.70	776.42	706.88	264.44	53.60
	5:1:2 FTB	1114.49	1678.51	647.76	284.73	129.07	47.05
	6:2 FTSA	380.23	854.85	481.55	647.14	182.19	33.33
	8:2 FTSA	295.75	227.70	42.98	42.38	9.04	3.00
	5:3 FTB	133.55	288.70	103.56	43.68	23.90	7.98
	PFHxS	127.24	337.74	189.15	229.57	55.49	11.26
	PFOA	78.51	157.44	52.85	63.71	12.54	2.85
	PFPeA	64.81	328.99	220.18	1003.56	215.95	51.77
	5:3 FTCA	42.50	40.24	8.67	9.11	0.00	0.00
	PFHxSAm	35.33	40.69	12.89	7.73	0.86	0.23
	PFOSAm	32.46	15.76	3.90	3.63	0.85	0.27
	PFHxA	23.77	158.79	137.19	417.69	81.84	18.04
	PFNA	23.21	16.18	6.50	7.02	0.00	0.00
	PFHpA	20.44	87.23	61.42	122.87	20.39	4.57
	PFHpS	18.12	27.47	8.78	7.94	1.88	0.40
	PFBA	13.84	46.17	23.60	196.87	30.37	2.95
	PFNS	10.69	5.59	1.78	1.83	0.70	0.00
	PFHxSAm-Pr-DiMeAm	9.38	15.56	3.02	3.37	5.24	1.00
	7:3 FTCA	9.09	4.82	1.01	1.22	0.00	0.46
	6:2 FTSAm-Pr-DiMeNO	9.08	14.48	2.39	2.21	0.00	0.00
	PFPeS	5.89	54.72	42.00	79.37	14.18	2.72
	PFDA	5.43	2.57	0.00	0.00	0.00	0.00
	PFDS	4.14	1.94	0.48	0.62	0.26	0.00
	PFDoDS	3.41	1.91	0.50	0.70	0.00	0.00
	PFBS	1.87	15.36	16.18	64.29	10.43	2.34
	PFUnDA	1.08	0.00	0.00	0.00	0.00	0.00
	PFDoDA	0.84	0.00	0.00	0.00	0.00	0.00
Semiquantified	6:2 FTSO-(2')OHPr-TriMeAm	2287.23	1483.84	146.58	171.38	135.02	18.31
	6:2 FTSy-(2')OHPr-TriMeAm	1880.23	820.60	66.81	107.66	14.87	4.05
	7:1:2 FTB	877.92	422.99	57.44	42.16	21.34	7.23
	9:1:2 FTB	641.06	255.54	29.36	41.02	49.67	9.18
	6:2 FTSAm	567.26	361.05	70.35	50.50	6.16	1.35
	6:2 FTSAm-Pr-DiMeAm	203.42	910.59	147.39	94.05	55.37	5.33
	7:3 FTB	190.68	128.42	16.60	10.34	5.63	1.05
	9:3 FTB	132.99	47.18	5.48	7.59	9.90	1.48
	PFOSAm-Pr-TriMeAm	87.71	32.74	4.46	5.85	6.31	0.56
	11:1:2 FTB	85.20	46.22	4.25	7.92	12.54	3.61

6:2/6:2 FTSAm dimer	72.35	56.44	7.00	10.43	2.64	0.28
6:2 FTTh-(2')OHPr-TriMeAm	65.67	102.62	39.64	54.81	109.83	10.52
8:2 FTSy-(2')OHPr-TriMeAm	61.21	15.58	1.41	2.92	0.62	0.13
8:2 FTSO-(2')OHPr-TriMeAm	59.10	35.28	2.64	3.96	4.38	0.92
6:2 FTSAm-*N*-Me-*N*-PrA	52.37	7.44	1.34	1.50	0.25	0.00
SF_5-PFOS	41.40	21.99	3.91	5.08	1.49	0.18
6:2 FTSAm-Pr-MeAm	41.25	302.93	60.27	30.28	7.35	0.70
PFOSAm-Pr-DiMeAm	31.13	89.28	13.46	13.89	14.87	1.58
SF_5-PFNS	25.35	13.75	2.49	3.17	0.82	0.09
6:4 FTB	25.33	28.23	5.15	2.74	1.61	0.43
11:3 FTB	21.90	11.77	1.10	1.97	3.06	0.87
13:1:2 FTB	19.71	14.81	1.49	3.13	5.25	1.08
8:2 FTSy-PrA	17.47	4.73	0.70	1.35	0.00	0.00
PFPeSAm	16.83	59.35	14.09	6.95	0.41	0.07
K-6:2 FTSA	15.87	74.21	23.40	28.41	4.22	0.54
6:2 FTSy-PrA	15.44	18.05	1.33	0.77	0.00	0.00
6:2 FTSAm-PrA	15.32	1.78	0.36	0.38	0.00	0.00
6:2/8:2 FTSAm dimer	13.93	0.68	0.15	0.77	0.08	0.00
PFOSAm-EtA	13.69	2.19	0.35	0.48	0.00	0.00
Cl-PFOS	13.12	10.25	2.28	1.95	0.29	0.00
6:2 FTB	12.91	18.88	4.81	1.91	0.67	0.24
8:2 FTSAm-Pr-DiMeAm	12.44	5.12	0.66	0.58	0.82	0.00
PFOSAm-Pr-B	11.96	5.68	0.57	0.46	0.41	0.00
8:4 FTB	11.53	4.04	0.50	0.65	0.81	0.09
K-8:2 FTSA	11.29	9.38	2.14	1.60	0.00	0.00
PFHxSAm-Pr-TriMeAm	10.56	12.26	1.66	1.33	0.30	0.08
PFBSAm	10.49	69.51	21.14	15.69	0.99	0.14
6:2 FTSAm-U-Pr-DiMeAm	10.13	25.12	8.94	10.18	5.67	0.67
8:2 FTB	10.00	4.37	0.62	0.62	0.26	0.07
U-8:2 FTSA	9.75	7.04	0.00	0.00	0.00	0.00
U-PFOS	8.09	19.85	5.32	4.57	0.78	0.12
8:2 FTTh-(2')OHPr-TriMeAm	7.20	10.72	2.75	4.48	10.45	1.88
7:1:3 FTB	7.06	2.29	0.18	0.24	0.12	0.00
8:2 FTSAm-Pr-B	6.57	1.71	0.22	0.25	0.22	0.16
H-PFOS	6.33	9.62	3.27	3.31	0.68	0.00
U-6:2 FTSA	6.18	23.81	8.91	10.65	1.44	0.00
PFHxSAm-Pr-B	5.74	8.55	1.41	0.96	0.43	0.00
4:2 FTSy-(2')OHPr-TriMeAm	5.68	2.28	0.00	0.00	0.00	0.00
PFTriDS	5.58	3.63	0.75	1.14	0.60	0.00
4:2 FTTh-(2')OHPr-TriMeAm	5.39	18.19	0.00	1.89	0.99	0.23
SF_5-U-PFOS	5.33	2.51	0.40	0.39	0.00	0.00
8:2 FTSAm	5.27	1.15	0.29	0.36	0.05	0.00
PFHxSAm-EtA	5.05	4.70	0.77	1.00	0.25	0.05
10:2 FTSA	4.85	1.82	0.20	0.45	0.00	0.00
H-PFDS	4.80	3.51	1.22	1.00	0.31	0.00

PFUnDS	4.53	1.77	0.49	0.66	0.22	0.00
13:3 FTB	4.46	2.69	0.30	0.62	1.04	0.21
OH-8:2 FTSA	4.11	2.56	0.58	0.52	0.00	0.00
U-E-PFNS/K-PFOS	3.40	4.39	1.14	0.97	0.19	0.04
5:1:3 FTB	3.36	2.85	0.34	0.00	0.00	0.00
SF_5-PFDS	3.17	1.72	0.41	0.48	0.00	0.00
U-PFDoDS	3.14	0.00	0.30	0.39	0.00	0.00
SF_5-U-PFNS	3.08	1.45	0.29	0.34	0.00	0.00
8:2 FTSy-Pr-Ad-(5',5')DiMeEtSA	2.71	10.02	0.62	0.27	0.03	0.00
U-PFUnDS	2.65	1.06	0.20	0.29	0.10	0.00
U-PFTriDS	2.47	0.00	0.00	0.00	0.00	0.00
10:2 FTB	2.26	0.92	0.12	0.00	0.21	0.00
U-E-PFTriDS/K-PFDoDS	2.04	1.06	0.00	0.00	0.00	0.00
PFOSyA	2.04	0.31	0.12	0.24	0.49	0.08
OH-6:2 FTSA	1.99	15.69	5.62	11.77	1.01	0.13
10:4 FTB	1.65	0.74	0.06	0.00	0.26	0.00
9:1:3 FTB	1.61	0.40	0.00	0.09	0.00	0.00
PFHxSAm-PrSA	1.52	5.11	1.09	1.14	0.21	0.00
8:2 FTSAm-Pr-DiMeNO	1.32	0.70	0.16	0.26	0.00	0.00
SF_5-PFHpS	1.23	0.70	0.13	0.15	0.00	0.00
U-PFDS	1.23	0.69	0.00	0.00	0.00	0.00
PFHxSAm-*N*-PrSA-*N*-Pr-DiMeAm	1.21	4.28	0.77	0.57	0.22	0.00
12:2 FTSA	1.17	0.37	0.06	0.12	0.00	0.00
14:2 FTSA	1.09	0.36	0.07	0.11	0.00	0.00
PFBSAm-Me	1.08	3.01	0.35	0.15	0.00	0.00
4:4 FTB	1.03	2.76	0.58	0.35	0.00	0.00
U-PFTeDS	1.03	0.00	0.00	0.00	0.00	0.00
PFHpSAm	1.02	0.60	0.12	0.00	0.00	0.00
6:2 FTSy-Pr-Ad-(5',5')DiMeEtSA	1.00	6.28	0.75	0.64	0.04	0.00
10:2 FTSAm	0.88	0.14	0.08	0.06	0.00	0.00
U-PFNS	0.67	0.55	0.00	0.00	0.00	0.00
PFOSAm-Me	0.59	0.26	0.00	0.04	0.00	0.00
6:2 FTSO-Pr-Ad-(5',5')DiMeEtSA	0.58	0.34	0.03	0.04	0.00	0.00
10:2 FTSy-Pr-Ad-(5',5')DiMeEtSA	0.42	1.25	0.13	0.13	0.00	0.00
PFPrS	0.21	2.25	2.24	23.17	2.54	0.50
U-PFHxS	0.18	2.38	2.06	4.33	0.99	0.00
12:2 FTSy-Pr-Ad-(5',5')DiMeEtSA	0.18	0.20	0.03	0.00	0.00	0.00
U-E-PFHpS/K-PFHxS	0.08	0.91	0.47	0.99	0.13	0.02
U-E-PFOS/K-PFHpS	0.07	0.37	0.14	0.15	0.02	0.00
U-PFHpS	0.05	0.38	0.17	0.19	0.00	0.00
PFBSAm-Pr-B	0.00	0.13	0.05	0.00	0.00	0.00

J. Extractable organofluorine

Table S12. Mean extractable organofluorine ($\overline{\mathbf{EOF}}$) and relative standard deviation (RSD) of combined extracts from sequential extraction with methanol only (MeOH) and methanol with 0.4 M NH_4Ac (MeOH+NH_4Ac) in triplicates for soil (A, B, C) and extraction blank. The limit of quantification (LOQ) was 0.08 µg F/g.

**Sample**	$\overline{\mathbf{EOF}}$ **(µg F/g)**	**RSD (%)**
Extract MeOH A	4.12	0.23
Extract MeOH B	4.14	0.02
Extract MeOH C	4.02	1.67
Extract blank MeOH	0.09	4.87
Extract MeOH+NH_4Ac A	7.53	2.38
Extract MeOH+NH_4Ac B	7.91	2.31
Extract MeOH+NH_4Ac C	8.08	1.11
Extract blank MeOH	0.09	5.43

K. Extraction recovery

Table S13. Mean extraction recovery and RSD determined from spike experiment on standard soil (LUFA SP6S, ~1.55% organic carbon), as well as MEs for compounds with available internal standard. ME was calculated according to equation 4 (n.a. = not applicable). Note that for substances where the ME could be calculated (blue font) the extraction recovery was corrected for ME.

Standard	$\overline{\text{Recovery}}$ (%)	RSD (%)	ME (%)
PFBA	122.50	2.79	-92.36
PFPeA	143.96	5.2	-81.91
PFHxA	138.96	7.59	-21.33
PFHpA	148.89	9.03	-11.66
PFOA	139.63	3.77	-32.19
PFNA	144.26	7.46	-6.6
PFDA	173.05	2.01	-20.59
PFBS	138.02	6.01	-42.72
PFPeS	103.79	6.41	25.08
PFHxS	181.52	3.79	-14.4
PFHpS	135.70	7.05	n.a.
PFOS	144.88	8.13	6.96
PFHxSAm	126.74	13.59	n.a.
PFOSAm	124.77	1.82	n.a.
PFOSAm-*N*-Et-*N*-EtA	84.14	10.34	n.a.
PFHxSAm-Pr-DiMeAm	105.17	10.03	n.a.
5:3 FTCA	61.51	7.1	n.a.
U-6:2 FTCA	70.17	4.15	n.a.
U-8:2 FTCA	92.35	4.02	n.a.
6:2 FTSA	296.74	10.48	n.a.
8:2 FTSA	175.65	1.92	n.a.
5:1:2 FTB	28.53	21.77	n.a.
5:3 FTB	22.54	6.61	n.a.
6:2 FTSAm-Pr-B	47.75	30.24	n.a.
6:2 FTSAm-Pr-DiMeNO	176.86	1.9	n.a.

L. Depth distribution

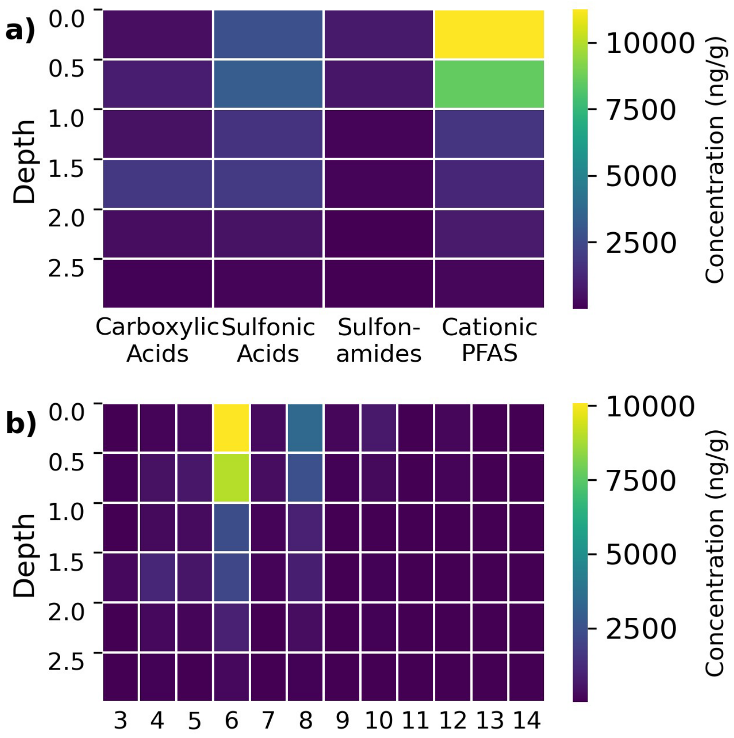

Figure S6. Depth distribution of summed concentrations segmented by a) ionization class and b) chain length.

Table S14. Water content.

Depth (m)	Water content (% w/w)
0-0.5	12.6
0.5-1	33.7
1-1.5	19.9
1.5-2	51.3
2-2.5	32.8
2.5-3	14.1

References

(1) Wikimedia Commons. Karte Deutschland. 2021. https://commons.wikimedia.org/wiki/File:Karte_Deutschland.svg (accessed 02.07.2024).
(2) Cao, D.; Schwichtenberg, T.; Duan, C.; Xue, L.; Muensterman, D.; Field, J. Practical Semiquantification Strategy for Estimating Suspect Per- and Polyfluoroalkyl Substance (PFAS) Concentrations. *J Am Soc Mass Spectrom* **2023**, *34* (5), 939-947. DOI: 10.1021/jasms.3c00019
(3) Pu, S.; McCord, J. P.; Bangma, J.; Sobus, J. R. Establishing performance metrics for quantitative non-targeted analysis: a demonstration using per- and polyfluoroalkyl substances. *Anal Bioanal Chem* **2024**, *416* (5), 1249-1267. DOI: 10.1007/s00216-023-05117-4
(4) Lauria, M. Z.; Sepman, H.; Ledbetter, T.; Plassmann, M.; Roos, A. M.; Simon, M.; Benskin, J. P.; Kruve, A. Closing the Organofluorine Mass Balance in Marine Mammals Using Suspect Screening and Machine Learning-Based Quantification. *Environ Sci Technol* **2024**, *58* (5), 2458-2467. DOI: 10.1021/acs.est.3c07220
(5) Malm, L.; Liigand, J.; Aalizadeh, R.; Alygizakis, N.; Ng, K.; Fro Kjaer, E. E.; Nanusha, M. Y.; Hansen, M.; Plassmann, M.; Bieber, S.; Letzel, T.; Balest, L.; Abis, P. P.; Mazzetti, M.; Kasprzyk-Hordern, B.; Ceolotto, N.; Kumari, S.; Hann, S.; Kochmann, S.; Steininger-Mairinger, T.; Soulier, C.; Mascolo, G.; Murgolo, S.; Garcia-Vara, M.; Lopez de Alda, M.; Hollender, J.; Arturi, K.; Coppola, G.; Peruzzo, M.; Joerss, H.; van der Neut-Marchand, C.; Pieke, E. N.; Gago-Ferrero, P.; Gil-Solsona, R.; Licul-Kucera, V.; Roscioli, C.; Valsecchi, S.; Luckute, A.; Christensen, J. H.; Tisler, S.; Vughs, D.; Meekel, N.; Talavera Andujar, B.; Aurich, D.; Schymanski, E. L.; Frigerio, G.; Macherius, A.; Kunkel, U.; Bader, T.; Rostkowski, P.; Gundersen, H.; Valdecanas, B.; Davis, W. C.; Schulze, B.; Kaserzon, S.; Pijnappels, M.; Esperanza, M.; Fildier, A.; Vulliet, E.; Wiest, L.; Covaci, A.; Macan Schonleben, A.; Belova, L.; Celma, A.; Bijlsma, L.; Caupos, E.; Mebold, E.; Le Roux, J.; Troia, E.; de Rijke, E.; Helmus, R.; Leroy, G.; Haelewyck, N.; Chrastina, D.; Verwoert, M.; Thomaidis, N. S.; Kruve, A. Quantification Approaches in Non-Target LC/ESI/HRMS Analysis: An Interlaboratory Comparison. *Anal Chem* **2024**, *96* (41), 16215-16226. DOI: 10.1021/acs.analchem.4c02902
